# A Mechanistic Model of Macromolecular Allocation, Elemental Stoichiometry, and Growth Rate in Phytoplankton

**DOI:** 10.3389/fmicb.2020.00086

**Published:** 2020-02-28

**Authors:** Keisuke Inomura, Anne Willem Omta, David Talmy, Jason Bragg, Curtis Deutsch, Michael J. Follows

**Affiliations:** ^1^School of Oceanography, University of Washington, Seattle, WA, United States; ^2^Department of Earth, Atmospheric and Planetary Sciences, Massachusetts Institute of Technology, Cambridge, MA, United States; ^3^Department of Microbiology, University of Tennessee, Knoxville, Knoxville, TN, United States; ^4^National Herbarium of New South Wales, The Royal Botanic Gardens and Domain Trust, Sydney, NSW, Australia

**Keywords:** phytoplankton, elemental stoichiometry, growth rate, macromolecule, photosynthesis, protein, RNA, nutrient storage

## Abstract

We present a model of the growth rate and elemental stoichiometry of phytoplankton as a function of resource allocation between and within broad macromolecular pools under a variety of resource supply conditions. The model is based on four, empirically-supported, cornerstone assumptions: that there is a saturating relationship between light and photosynthesis, a linear relationship between RNA/protein and growth rate, a linear relationship between biosynthetic proteins and growth rate, and a constant macromolecular composition of the light-harvesting machinery. We combine these assumptions with statements of conservation of carbon, nitrogen, phosphorus, and energy. The model can be solved algebraically for steady state conditions and constrained with data on elemental stoichiometry from published laboratory chemostat studies. It interprets the relationships between macromolecular and elemental stoichiometry and also provides quantitative predictions of the maximum growth rate at given light intensity and nutrient supply rates. The model is compatible with data sets from several laboratory studies characterizing both prokaryotic and eukaryotic phytoplankton from marine and freshwater environments. It is conceptually simple, yet mechanistic and quantitative. Here, the model is constrained only by elemental stoichiometry, but makes predictions about allocation to measurable macromolecular pools, which could be tested in the laboratory.

## Introduction

Phytoplankton are responsible for the majority of photosynthesis in the ocean (Field et al., 1998) and more than half in lakes (Vadeboncoeur et al., 2002). The elemental stoichiometry of phytoplankton varies significantly through acclimation and adaptation (Quigg et al., 2003, 2011; Finkel et al., 2016), modulates fitness in different environments (Deutsch and Weber, 2012), global ocean carbon storage (Galbraith and Martiny, 2015), and the nutrition of higher trophic levels (Mitra et al., 2007). Population growth rates of phytoplankton depend on resource availability (Caperon and Meyer, 1972a,b; Paasche, 1973; Laws and Bannister, 1980; Pedersen and Borum, 1996; Xu et al., 2010) and also vary through acclimation and adaptation (Falkowski and Owens, 1980; Levasseur et al., 1993; Litchman et al., 2002, 2003; Collos et al., 2005; Litchman and Klausmeier, 2008; Van Mooy et al., 2009; Lewis et al., 2019). The environmentally dependent growth rate of a population is an important component of its fitness and significant for ecological and biogeochemical modeling.

**Figure 1:**
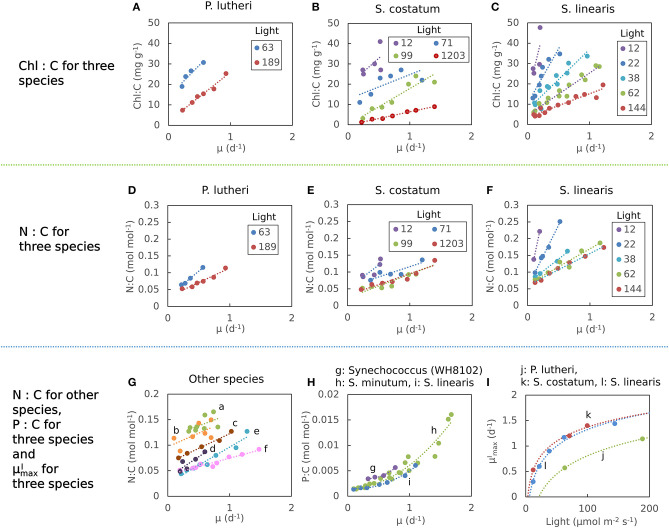
Compiled laboratory data of growth rate and light dependence of chlorophyll and elemental stoichiometry, and light dependence of $\mu_{max}^{I}$: nutrient replete growth rate. Data are all from chemostat cultures where the growth rate, μ, is controlled by the dilution rate, *D*. Here, illustrated N:C data were all under N limitation and P:C were all under P limitation. Points represent the original data and curves represent regression lines. **(A**–**C)** Chlorophyll:C for various light intensities for *Pavlova lutheri* (Chalup and Laws, 1990), *Skeletonema costatum* (Sakshaug and Andersen, 1989), and *Synechococcus linearis* (Healey, 1985). Legends indicate light intensities in μmol m^−2^ s^−1^. **(D**–**F)** N:C for various light intensities for the same organisms as **(A**–**C)**, respectively. Legend values are light intensities as in **(A**–**C)**. **(G)** N:C for other organisms. (a) *Synechococcus* (WH8102) (Garcia et al., 2016). (b) *Synechococcus* (WH7803) (Liu et al., 1999). (c) *Coccochloris stagnina* (Caperon and Meyer, 1972a). (d) *Thalassiosira pseudonana* (Claquin et al., 2002). (e) *Dunaliella tertiolecta* (Caperon and Meyer, 1972a). (f) *Monochrysis lutheri* (Caperon and Meyer, 1972a). **(H)** P:C for three organisms. (g) *Synechococcus* (WH8102) (Garcia et al., 2016). (h) *Selenastrum minutum* (Elrifi and Turpin, 1985). (i) *Synechococcus linearis* (Healey, 1985). In **(G,H)** a light intensity varied between experiments. **(I)** $\mu_{max}^{I}$- light relationships for three organisms as in **(A**–**C)**. (j) *Pavlova lutheri* (Chalup and Laws, 1990). (k) *Skeletonema costatum* (Sakshaug and Andersen, 1989). (l) *Synechococcus linearis* (Healey, 1985). For *Synechococcus linearis*, only data points where the limiting nutrient was fully consumed are plotted (this applies to other figures).

The elemental stoichiometry and growth rate of phytoplankton are not independent. Robust qualitative relationships between growth rate, elemental stoichiometry, and resource availability are evident in controlled laboratory cultures spanning wide taxonomic and allometric ranges. We illustrate this in Figure 1, Supplementary Figure 1 with data compiled from published, continuous culture laboratory studies of 12 species, including marine, freshwater, prokaryotic, and eukaryotic phytoplankton. In all cases, at a fixed irradiance, Chl:C (chlorophyll per carbon) increases linearly with growth rate, μ (Laws and Bannister, 1980; Healey, 1985; Sakshaug and Andersen, 1989; Chalup and Laws, 1990), and both the slope and the intercept increase as the irradiance declines (Figures 1A–C) (quantitative fits with *R*^2^ values in Supplementary Table 1). Similarly, cellular N:C (nitrogen:carbon) increases linearly with growth rate (Caperon and Meyer, 1972a; Laws and Caperon, 1976; Laws and Bannister, 1980; Healey, 1985; Sakshaug and Andersen, 1989; Chalup and Laws, 1990; Figures 1D–G) and its slope and intercept both increase with decreasing photon flux (Healey, 1985; Sakshaug and Andersen, 1989; Chalup and Laws, 1990; Figures 1D,F, *R*^2^ values in Supplementary Table 2). In contrast, the cellular P:C (phosphorus:carbon) increases non-linearly with growth rate (Figure 1H).

**Figure 2:**
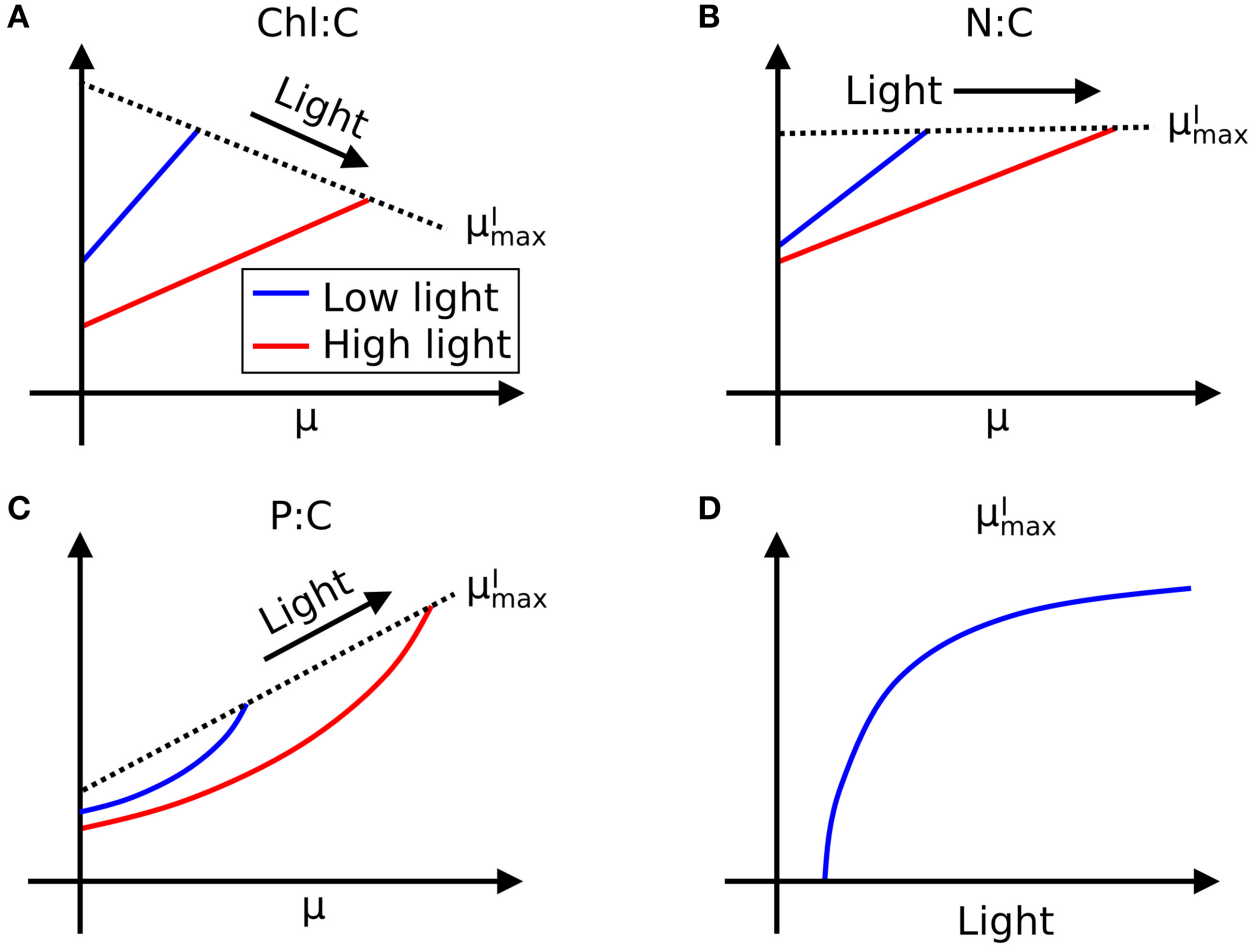
Illustration of general trends in laboratory data in chemostat culture studies. Growth rate (μ) and light dependence of **(A)** Chl:C, **(B)** N:C under N limitation, and **(C)** P:C under P limitation, respectively, and **(D)** $\mu_{max}^{I}$–light relationships. Whereas, Chl:C and N:C are described by linear curves, P:C has non-linear relationships with μ. Black dotted lines represent $\mu_{max}^{I}$.

Here we define $\mu_{max}^{I}$ as maximum growth rate for a given light intensity. $\mu_{max}^{I}$ can be also considered as a nutrient replete growth rate. In the chemostat culture, as the dilution rate increases, the rate of nutrient input increases. Despite the increased rate, when the dilution rate is above $\mu_{max}^{I}$, the cells are flushed away, since cellular growth cannot increase further. Thus, $\mu_{max}^{I}$ is indicated by the termination of the linear increase in Chl:C and N:C, and the termination of the non-linear increase in P:C with μ (Figures 1A–H, and indicated schematically by the dotted line in Figures 2A–C). In Figure 1I, we plot $\mu_{max}^{I}$ as a function of light intensity revealing the typical saturation of growth rate at high light intensities (Healey, 1985) (we note that none of the illustrated experiments were in a regime of photo-inhibition). Figures 1A–I, Supplementary Figure 1 thus reveal a set of robust qualitative relationships between light intensity, growth rate, and the elemental stoichiometry of diverse phytoplankton under steady-state growth conditions (summarized schematically in Figure 2).

The common patterns in Figure 1 reflect the fact that, despite the diversity of species represented, there are shared physiological underpinnings. The elemental stoichiometry of a cell depends on the relative abundances of the macromolecules from which it is composed and they, in turn, are linked to environment and physiological state (Sterner and Elser, 2002). The C:N:P stoichiometry of phytoplankton can be largely accounted for by the sum of contributions from a handful of major macromolecular components: protein, pigment, carbohydrate, lipid, DNA, RNA, and storage molecules (Liefer et al., 2019) each of which has a distinct elemental stoichiometry (see Table 1). For example, proteins are relatively rich in nitrogen so increasing the cellular allocation to protein typically raises cellular N:C (Sterner and Elser, 2002; Klausmeier et al., 2004). The broad-brush response of the macromolecular allocation of phytoplankton to changes in environmental factors is common across broad taxonomic groupings; for example in laboratory studies of nitrogen starvation in four marine species (Liefer et al., 2019) and with changing temperature and growth rate amongst a wide variety of freshwater phytoplankton (Fanesi et al., 2017, 2019).

**Table 1:** Elemental stoichiometry of some macromolecules.

**Molecule**	**C:N:P**	**Explanation**
Chlorophyll	55:4:0	Chlorophyll A
Protein	3.82:1:0	Average value based on (Brown, 1991)
RNA	9.5:3.78:1	Based on CG = 0.563: *Synechococcus spp*.^*^
DNA	9.72:3.78:1	Based on CG = 0.563: *Synechococcus spp*.^*^
P lipid	40:0:1	Phosphatidylglycerol with C16 fatty acids
C store	1:0:0	Carbohydrate and non-phospholipid
N store	2:1:0	Cyanophycin
P store	0:0:1	Polyphosphate

* *GC% [http://www.ncbi.nlm.nih.gov/genome/13522 (accessed December 13, 2018)]*.

Models of phytoplankton physiology have sought to relate growth rate (related to fitness) and elemental stoichiometry (related to biogeochemical impacts) to external resource availability (Riley, 1946; Monod, 1949), internal stores of resources (Caperon, 1968; Droop, 1968), and the internal allocation between functional pools and storage molecules (Shuter, 1979; Geider et al., 1998; Kooijman, 2010). Recent models also explicitly represent trade-offs associated with allocation of the resource and proteome (Bonachela et al., 2013; Burnap, 2015; Smith et al., 2016; Reimers et al., 2017; Chen and Smith, 2018; Faizi et al., 2018; Jahn et al., 2018; Faizi and Steuer, 2019). We provide a more comprehensive review of published physiological models in Supplementary Text. The model presented here also aims to explicitly connect growth rate, elemental stoichiometry, and environmental conditions. It is based on the allocation of resources between and within the major macromolecular pools. We seek to frame the model in terms of measurable (rather than abstracted) pools, to provide interpretations of observed laboratory relationships, and to keep the model efficient and simple for practical applications.

## Model Description

**Figure 3:**
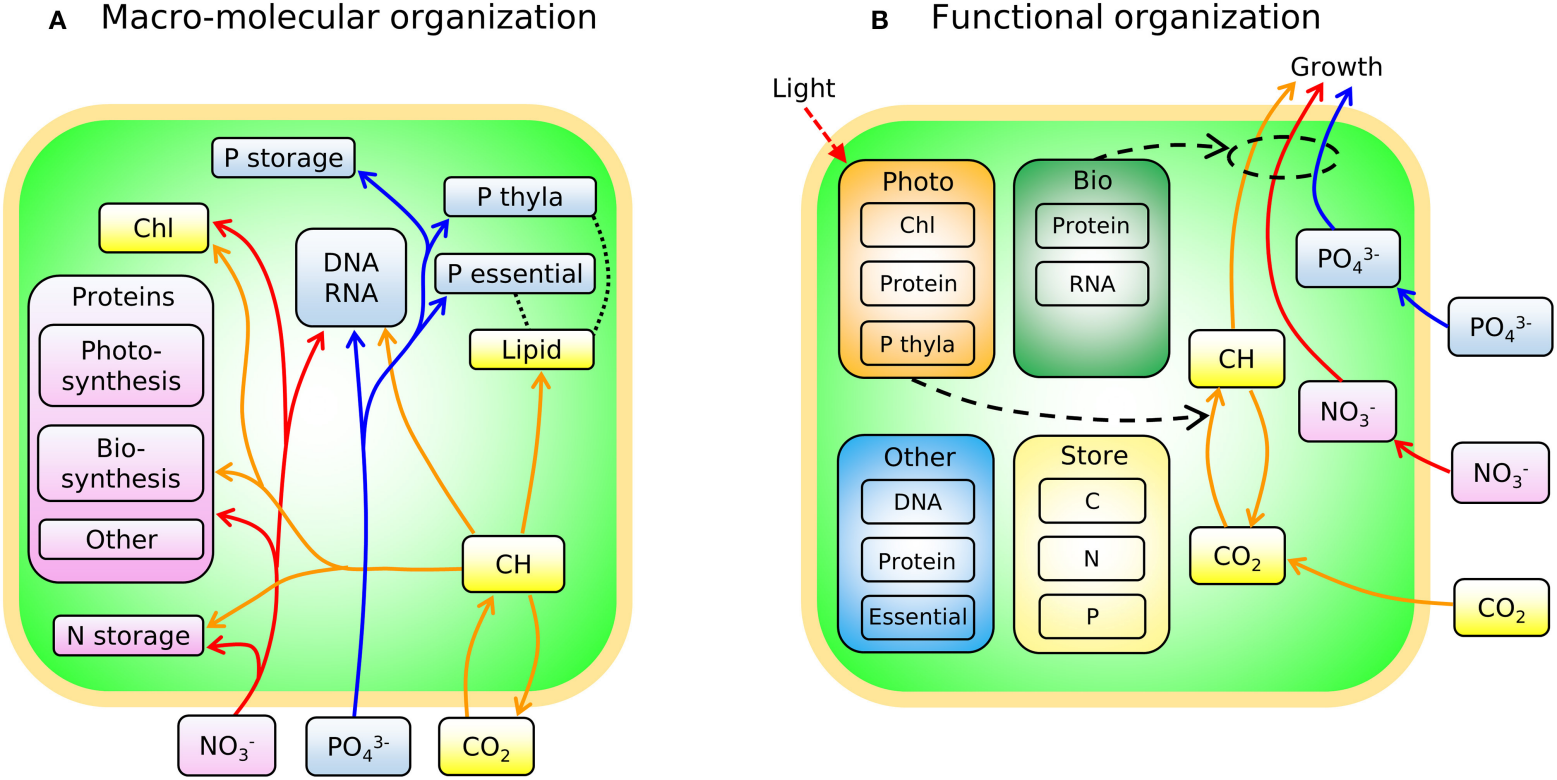
Schematic of the two different views of the model: CFM-Phyto. **(A)** Allocation of C, N, and P to key macromolecular pools. Orange outline, cell membrane layers; green background is cytoplasm. Arrows represent elemental fluxes; yellow, C; red, N; blue, P. Colors in boxes represent elements that are influenced by each molecule most. Yellow: C. Red: N. Blue: P. Black dotted lines indicate possible intramolecular associations of C and P. Orange outline, cell membrane layers; green background, cytoplasm; *Chl*, chlorophyll; *thyla*, thylakoid; *CH*, carbohydrate. **(B)** Simple view of macromolecular allocation grouped into four different functions; *Bio*, biosynthetic molecules; *Photo*, photosynthetic molecules; *Other*, other constant molecules; *Store*, storage molecules. *Bio* affects growth rates and *Photo* affects photosynthesis rates. Essential in *Other* indicates essential lipids and carbohydrates. Black dashed arrows indicate positive influences. Red dashed arrow represents light.

In Figure 3A we sketch the broad-brush allocation of C, N, and P to key macromolecular pools in the phytoplankton model (Cell Flux Model of Phytoplankton: CFM-Phyto). Cells also allocate resources within the macromolecular pools. For example, lipids incorporate lipid membranes and lipids storage molecules (Shifrin and Chisholm, 1981; Lengeler et al., 1999). The protein pool includes enzymes devoted to hundreds of reactions which may be coarse-grained into several major categories including those related to light-harvesting and electron transport, and those related to biosynthesis, growth, and reproduction (Figure 3A). Recent proteomic analyses are quantifying broad-brush protein allocation (McKew et al., 2013, 2015; Christie-Oleza et al., 2017; Jahn et al., 2018; Zavřel et al., 2019) in ways which connect to such coarse-grained models (Scott et al., 2010; Burnap, 2015; Reimers et al., 2017; Faizi et al., 2018; Faizi and Steuer, 2019). Recent studies have revealed that a large and highly variable fraction of phytoplankton proteome is devoted to light-harvesting and electron transport (Jahn et al., 2018; Zavřel et al., 2019) and this investment increases as light intensity decreases. The macromolecular pools identified in Figure 3A are potentially measurable (Scott et al., 2010; McKew et al., 2013, 2015; Jahn et al., 2018; Fanesi et al., 2019; Zavřel et al., 2019), which is useful for testing and calibrating. At the same time, it is useful to recast these macromolecular pools into “functional units.” For example, in low light, cyanobacteria allocate to increase light harvesting proteins, but also pigments and lipids in the thylakoid membrane. We illustrate this re-organization in Figure 3B. Allocation-based models of phytoplankton populations have abstracted the system at this level (Shuter, 1979; see Supplementary Text), providing mechanistic representations of trade-offs but which are more difficult to directly constrain from observations. The relationship between Figures 3A,B indicates how the two perspectives are compatible. The model we outline below is developed in terms of the measurable pools indicated in Figure 3A, but interpreted in terms of their aggregated, functional allocation as depicted in Figure 3B.

In the following sections, we outline an idealized, allocation-based model of phytoplankton physiology and growth rate under a range of resource conditions (N, P, light). We show that the observed relationships between Chl:C, N:C, P:C growth rate and light (Figure 1, Supplementary Figure 1; summarized schematically in Figure 2) can be quantitatively modeled by understanding carbon allocation between and within the major macromolecular pools. By considering the allocation of nitrogen, we find an interpretation for the linear relationship of N:C with growth rate and its dependence on light intensity. By relating allocation of phosphorus to the rate of biosynthesis, we model and interpret the non-linear relationship between P:C and growth rate. The model is developed with particular reference to Healey's study of *Synechococcus linearis*, a freshwater cyanobacterium (Healey, 1985; Figures 1C,F,i,l), which provides a comprehensive set of constraints on elemental stoichiometry at multiple growth rates and light intensities, under both N and P limitation. In the experimental data, there are no direct constraints on macromolecular allocation, so we infer the latter through combination of observed elemental stoichiometry and model structure and discuss the inferred macromolecular allocation with reference to other published studies. While the model has been developed by exploiting the comprehensive data set of Healey (1985), the physiology of allocation at this level is common across taxa and it can be fitted to data from other phytoplankton, as we also illustrate.

## Modeled Macromolecular Composition of the Cell

### Carbon Allocation

Laboratory studies have shown that almost all the cellular carbon in phytoplankton is accounted for by the major macromolecular pools: proteins (*C*_*Pro*_), chlorophyll and other pigments (*C*_*Chl*_), nucleic acids (*C*_*Nuc*_), carbohydrates (*C*_*Carb*_), and lipids (*C*_*Lip*_) (Anderson, 1995; Liefer et al., 2019). The carbon quota of a phytoplankton cell, *C*_*Cell*_ (mol C cell^−1^) is thus defined as the sum of these components along with carbon associated with nitrogen storage molecules, *C*_*Nsto*_:


(1) $$\begin{array}{l} C_{Cell}=C_{Pro}+C_{Nuc}+C_{Lip}+C_{Carb}+C_{Chl}+C_{Nsto} \end{array}$$

While chlorophyll is a relatively minor contribution in this regard, it provides a constraint on the light harvesting capacity of the cells and is routinely measured. Here we neglect the contribution from intra-cellular, dissolved metabolites which are typically minor [e.g., ~4% of cellular dry weight in *E. Coli* (Lengeler et al., 1999) and predicted to be only ~1% as inorganic ions in *Synechococcus elongatus* (PCC 7942) (Reimers et al., 2017)].

Elemental variations referenced to carbon (C:C, N:C, P:C) present clearer relationships with μ and light than cellular quotas (Caperon and Meyer, 1972a; Laws and Bannister, 1980; Elrifi and Turpin, 1985), so we define carbon normalized macromolecular composition by dividing both sides of Equation (1) by *C*_*Cell*_:


(2) $$\begin{array}{l} 1=Q_{C}^{Pro}+Q_{C}^{Nuc}+Q_{C}^{Lip}+Q_{C}^{Carb}+Q_{C}^{Chl}+Q_{C}^{Nsto} \end{array}$$

where $Q_{C}^{i}$ are ratios with units of (mol C mol C^−1^). Proteins account for a large fraction of the carbon and nitrogen in a phytoplankton cell (Anderson, 1995; Geider and La Roche, 2002).

Recent quantitative proteomics studies revealed a coarse-grained reorganization of the proteome of *Synechocystis* in response to a changing light environment: an increase in photon flux drove an increase in growth rate with an associated downregulation of light harvesting machinery and upregulation of translational machinery (McKew et al., 2013; Jahn et al., 2018; Zavřel et al., 2019). This motivates the resolution of protein pools related to biosynthesis, $Q_{C}^{Pro-Bio},$ and photosynthesis, $Q_{C}^{Pro-Pho}$, the latter including contributions from light absorbing antennas, as well as proteins for photosystems and electron transport. Proteomic studies have shown that light-harvesting proteins contribute as much as 38% of the proteome of *Synechococcus* sp. WH7803 in culture (Christie-Oleza et al., 2017) and can vary considerably (Jahn et al., 2018; Zavřel et al., 2019). We seek to exploit these observed proteomic trade-offs and so resolve the allocation of protein into three functional pools:


(3) $$\begin{array}{l} Q_{C}^{Pro}=Q_{C}^{Pro-Pho}+Q_{C}^{Pro-Bio}+Q_{C}^{Pro-Other} \end{array}$$

We also resolve a fixed-size pool of “essential” proteins, $Q_{C}^{Pro-Other}$ which is necessary to close the cellular budget and notionally includes enzymes associated with essential metabolism (Jahn et al., 2018; Zavřel et al., 2019) and structure.

Nucleic acids include contributions from DNA and RNA:


(4) $$\begin{array}{l} Q_{C}^{Nuc}=Q_{C}^{DNA}+Q_{C}^{RNA} \end{array}$$

where the contribution from RNA is significantly more variable and related to growth rate; discussed in more detail below. Intracellular dissolved pools are not resolved since they generally represent <5% of the total cellular mass (Lengeler et al., 1999).

The lipid pool can be separated into three components. A large fraction of thylakoid membrane is lipid (~30%) (Kirchhoff, 2014), and we resolve a phospholipid fraction of it, $Q_{C}^{Plip-Thy}$, which also contributes significantly to the cellular phosphorus budget. $Q_{C}^{Lip-Sto}$ is a flexible pool of storage molecules (Werner, 1977; Shifrin and Chisholm, 1981), and $Q_{C}^{Lip-Other}\;$represents essential structural components of the cell membrane (Neidhardt et al., 1990; Lengeler et al., 1999), which we consider as a non-flexible pool. Hence, we resolve three lipid pools:


(5) $$\begin{array}{l} Q_{C}^{Lip}=Q_{C}^{Plip-Thy}+Q_{C}^{Lip-Sto}+Q_{C}^{Lip-Other} \end{array}$$

We represent the total cellular carbohydrate pool as the sum of two contributions: a flexible component, $Q_{C}^{Carb-Sto}$, representing storage (Shifrin and Chisholm, 1981; Deschamps et al., 2008; Dron et al., 2012) and the pool of essential carbohydrate metabolites (Lengeler et al., 1999; Michal, 1999), along with an “essential,” fixed carbohydrate contribution, $Q_{C}^{Carb-Other}$ (Harrison et al., 1990; Anderson, 1995; Biersmith and Benner, 1998):


(6) $$\begin{array}{l} Q_{C}^{Carb}=Q_{C}^{Carb-Sto}+Q_{C}^{Carb-Other} \end{array}$$

### Nitrogen Allocation

Cellular nitrogen (*N*_*Cell*_) is mostly associated with protein (*N*_*Pro*_) (Anderson, 1995; Liefer et al., 2019), along with contributions from RNA (*N*_*RNA*_), DNA (*N*_*DNA*_), chlorophyll (*N*_*Chl*_), and nitrogen storage (*N*_*Sto*_):


(7) $$\begin{array}{l} N_{Cell}=N_{Pro}+N_{RNA}+N_{DNA}+N_{Chl}+N_{Sto} \end{array}$$

Since N:C presents clearer relationships with μ and light than *N*_*Cell*_ (Caperon and Meyer, 1972a; Laws and Bannister, 1980; Elrifi and Turpin, 1985), we divide both sides of Equation (7) by *C*_*Cell*_:


(8) $$\begin{array}{l} N:C=Q_{N}^{Pro}+Q_{N}^{RNA}+Q_{N}^{DNA}+Q_{N}^{Chl}+Q_{N}^{Sto} \end{array}$$

where $Q_{N}^{i}$ are ratios with units of (mol N mol C^−1^). Each of the macromolecular pools has a distinct elemental stoichiometry (see Table 1), and carbon-normalized nitrogen quotas ($Q_{N}^{i}$, mol N mol C^−1^) are constructed accordingly. For example, the total nitrogen content of cellular protein, $Q_{N}^{Pro}=Q_{C}^{Pro}Y_{Pro}^{N:C}$, where $Y_{Pro}^{N:C}$ is the empirically-informed, average N:C of protein (see Table 1).

### Phosphorus Allocation

Nucleic acids (*P*_*RNA*_ and *P*_*DNA*_), phospholipids in the thylakoid membrane (*P*_*Thy*_), and storage compounds including polyphosphate (*P*_*Sto*_) are observed to account for most of the cellular phosphorus in phytoplankton and bacteria (Anderson, 1995; Lengeler et al., 1999; Table 1). Phosphorus may also be distributed in non-photosynthetic phospholipids and associated with other molecules (e.g., phosphorylation; Lengeler et al., 1999) which are here assumed to be in a fixed pool, *P*_*Other*0_. We also account for the flexible part of non-Thylakoid P-lipid in *P*_*Sto*_. Here we resolve phosphorus allocation to these distinct pools:


(9) $$\begin{array}{l} P_{Cell}=P_{RNA}+P_{DNA}+P_{Thy}+P_{Sto}+P_{Other0} \end{array}$$

We note that a full accounting for the phosphorus in phytoplankton has not been experimentally characterized to date (Moreno and Martiny, 2018; Liefer et al., 2019). As we have done for *C*_*Cell*_ and *N*_*Cell*_, we divide both sides of Equation (9) and obtain P:C:


(10) $$\begin{array}{l} P:C=Q_{P}^{RNA}+Q_{P}^{DNA}+Q_{P}^{Thy}+Q_{P}^{Sto}+Q_{P}^{Other0} \end{array}$$

where $Q_{P}^{i}$ are ratios with units of (mol P mol C^−1^). As for N:C, $Q_{P}^{i}$ is constructed according to the distinct elemental stoichiometry of each macromolecule (Table 1).

### Representing Relationships Between Macromolecular Pools and Rates

In addition to these statements of mass conservation and allocation, we must connect macromolecular allocation to rates. We do this assuming four mathematical representations of which three are well-supported by laboratory observations:

- *The per chlorophyll gross rate of photosynthesis, v*_*I*_ *(mol C (mol C in Chl)*^−1^ *d*^−1^*) is a saturating function of irradiance I (*μ*mol m*^−2^ *s*^−1^*)*. Following established models rooted in empirical observations and target theory (Cullen, 1990; Geider et al., 1998), we model photosynthesis as a function of light intensity:
  (11) $$\begin{array}{l} v_{I}\left(I\right)=v_{I}^{max}\left(1-e^{-A_{I}I}\right) \end{array}$$
  Here $v_{I}^{max}$ is the maximum photosynthesis rate per chlorophyll and *A*_*I*_ is a coefficient characterizing the absorption cross-section and turnover time of the photosynthetic unit (Cullen, 1990).
- *The components of the photosynthetic machinery, namely chlorophyll, light-related proteins, and the thylakoid phospholipids co-vary linearly*. In other words, the composition of the thylakoid apparatus remains constant but its amount per cell is varied with acclimation. Hence, the allocations to photosynthetic protein and thylakoid phospholipids are assumed linearly proportional to cellular chlorophyll content:
  (12) $$\begin{array}{l} Q_{C}^{Pro-Pho}=A_{Pho}\;Q_{C}^{Chl} \end{array}$$
  and
  (13) $$\begin{array}{l} Q_{P}^{Thy}=A_{Pho}^{P:Chl}\;Q_{C}^{Chl} \end{array}$$
  where *A*_*Pho*_ and $A_{Pho}^{P:Chl}$ are constants of proportionality. It is observed that the size of thylakoid membranes increases under low light in phytoplankton (Geider et al., 1996) and the chloroplasts of plants (Lichtenthaler et al., 1982). The thylakoid membranes are generally highly crowded with proteins (Folea et al., 2008; Kirchhoff et al., 2008; Kirchhoff, 2014) but how the fraction of proteins might change with growth conditions is less clear. Thus, we choose the simplest model and assume a fixed composition of the light harvesting apparatus.
- *Investment in biosynthetic protein is proportional to growth rate:*
  (14) $$\begin{array}{l} Q_{C}^{Pro-Bio}=\;A_{Bio}\;\mu \end{array}$$
  This is consistent with the observed linear increase in the investment in ribosomal proteins with growth rate in multiple cultures of *Synechocystis* (Jahn et al., 2018; Zavřel et al., 2019). Cultures of *Scenedesmus* sp. (Rhee, 1978), also show a near-linear relationship between protein-based nitrogen and growth rate under constant light. We note that $Q_{C}^{Pro-Bio}$ represents not only ribosomal proteins but includes those involved in synthesis of lipid and nucleic acids, C metabolism, and cell division.
- *The investment in RNA*, $Q_{P}^{RNA}$, *varies linearly with total protein content and with growth rate*. This relationship is derived from the observation that the RNA:protein ratio is linear with growth rate in phytoplankton (Nicklisch and Steinberg, 2009; Liefer et al., 2019), as is the case for heterotrophic bacteria (Bremer and Dennis, 1996; Scott et al., 2010). Thus, we model investment in RNA as
  (15) $$\begin{array}{l} Q_{P}^{RNA}=A_{RNA}^{P}\;\mu Q_{C}^{Pro}+Q_{P,min}^{RNA} \end{array}$$
  where $Q_{P,\;min}^{RNA}$ is the minimum RNA (mol P mol C^−1^), which occurs at zero growth rate. This relationship says that cells need more RNA to divide faster and/or to reproduce a higher cellular protein quota.

Relationships in (i), (iii), and (iv) above are directly supported by empirical data in the associated citations. The relationship between components of the light harvesting and photosynthesis machinery in (ii) is logical and simple, but unconfirmed by direct empirical data to our knowledge.

Using the above statements of mass conservation (Equations 12, 13) and representations of key relationships between fluxes and pools (Equations 14, 15), we model the observed dependencies of cellular stoichiometry (i.e., Chl:C, N:C, and P:C) on growth rate, light intensity and limiting factor, as well as the variation of maximum growth rate, $\mu_{max}^{I}$, under different light intensities. In the following sections we outline the model, making some approximations to the full equations presented above, in order to provide an illustrative and instructive discussion. A complete approach to solution of the model is presented in the Methods section and the solutions presented in all figures were generated using the un-approximated forms. Final equations of the un-approximated model are also summarized in tabular form in Supplementary Table 3. We frame our discussion of the model and its application by seeking to explain the trends identified in Figures 1, 2.

### Model Representation and Analysis

#### Why Does Chl:C Vary Linearly With Growth Rate?

Consider the rate of change of the cellular carbon quota, which is increased by photosynthesis, and reduced by division with population growth rate μ (d^−1^) and maintenance respiration rate *m* (d^−1^) (e.g., Geider et al., 1998; Pahlow and Oschlies, 2009):


(16) $$\begin{array}{l} \frac{dC_{Cell}}{dt}=v_{I}Q_{C}^{Chl}C_{Cell}-\left(1+E\right)C_{Cell}\;\mu \;-m\;C_{Cell} \end{array}$$

where $Q_{C}^{Cell}$ is cellular chlorophyll to cellular carbon ratio [(mol C in Chl) mol C^−1^], and *v*_*I*_ is the per chlorophyll rate of photosynthesis as defined in Equation (11). *E* is the respiratory cost of synthesis (moles C respired per mole C synthesized). *E* is estimated based on the production of biomass with stoichiometry of C_5_H_7_O_2_N_1_P_1/30_ using nitrate as the nitrogen source with energy transfer efficiency of 0.6 (Rittmann and McCarty, 2001). The assumed elemental stoichiometry is based on the suggested values of C:H:O:N (Rittmann and McCarty, 2001) and within the range observed in the laboratory experiments (Healey, 1985). Idealized models have typically assumed that *E* is proportional to cellular nitrogen content, assuming associated costs with nitrate reduction and protein synthesis (Laws and Wong, 1978; Geider et al., 1998; Pahlow and Oschlies, 2009). However, many aspects of metabolism consume ATP, including the synthesis of lipid and carbohydrate that contain little nitrogen (Lengeler et al., 1999; Michal, 1999) so here we assume the respiratory cost of biosynthesis is proportional to cellular carbon (i.e., *EC*_*Cell*_ in Equation 16).

In steady-state, the solution of Equation (16) anticipates the observed linear relationship between the cellular chlorophyll to carbon ratio ($Q_{C}^{Chl}=Chl\;:C$) and growth rate for any given photon flux, *I* (a similar relationship as in Laws and Bannister, 1980):


(17) $$\begin{array}{l} Q_{C}^{Chl}=A_{Chl}\left(I\right)\mu +B_{Chl}\left(I\right) \end{array}$$

where *A*_*Chl*_(*I*) = (1 + *E*)/*v*_*I*_(*I*) and *B*_*Chl*_(*I*) = *m*/*v*_*I*_(*I*). The predicted linear trend in Equation (17) is consistent with the data in Figure 1. We fitted Equation (17) to the data of Healey (1985) using the Metropolis-Hastings procedure (Metropolis et al., 1953; Hastings, 1970; Omta et al., 2017), a Markov Chain Monte Carlo method which is described in detail in Methods. The values of the parameters *m*, $v_{I}^{max},$ and *A*_*I*_ were optimized in the model-data fit, with single values for $v_{I}^{max}$ and *A*_*I*_ for all light levels.

**Figure 4:**
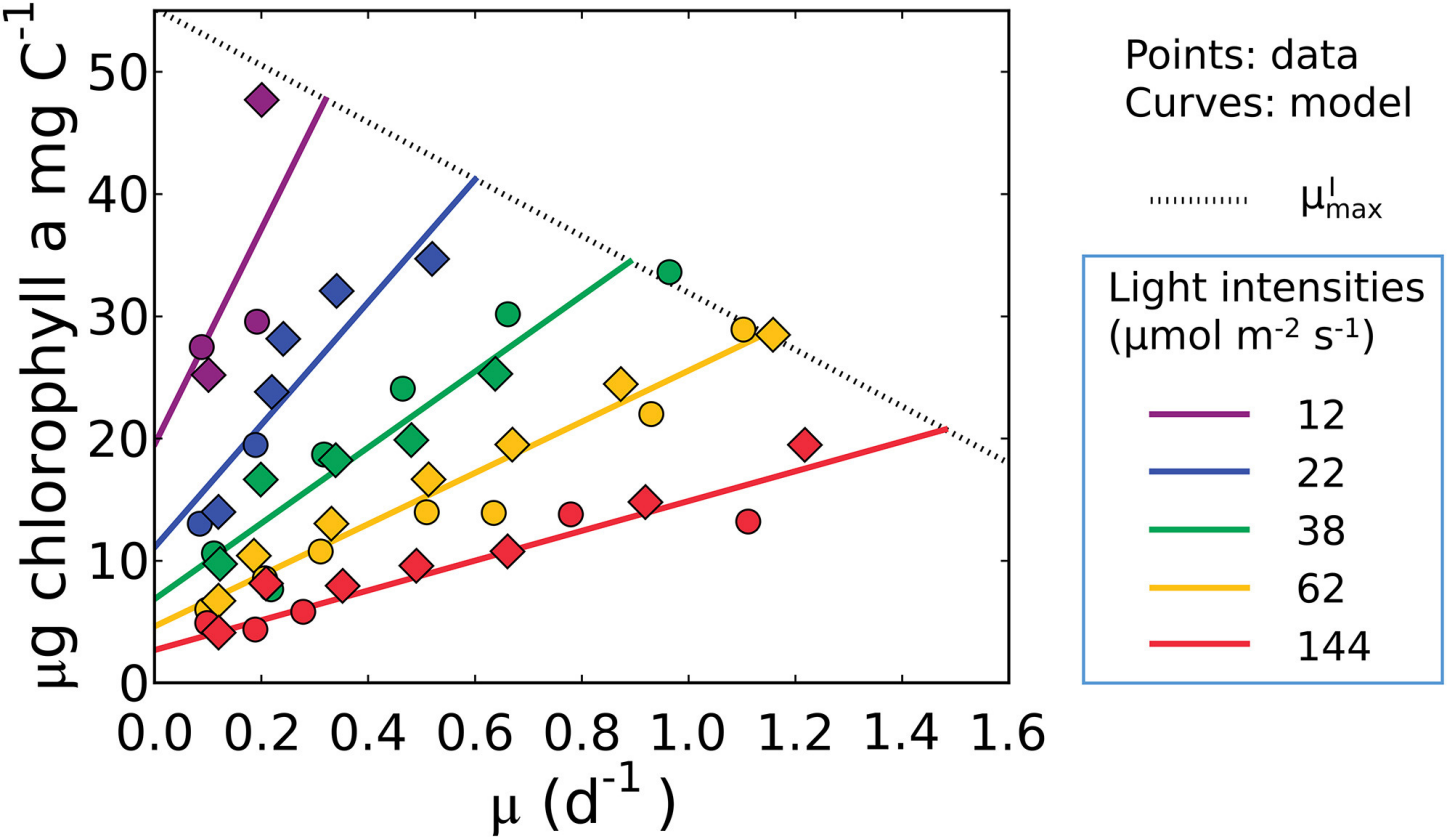
Model-data comparison of chlorophyll per C of *Synechococcus linearis* for various growth rates and light intensities. Curves: model solution (Equation 17). Points: data (Healey, 1985; circles, N limited; diamonds, P limited). Dotted line represents $\mu_{max}^{I}$ at various light intensities; high $\mu_{max}^{I}$ for higher light intensity. μ in the *x* axis represents growth rates (d^−1^). Legend values are light intensities (μmol m^−2^ s^−1^).

Equation (17), indicates that the cell must maintain pigments to sustain maintenance respiration even at a net zero growth rate (y-intercept). It predicts a linear relationship between Chl:C and μ, as well as an increase in both the slope and intercept of the Chl:C ratio with decreasing irradiance (Figure 4). The linear trend reflects the increased investment in light-harvesting machinery to maintain the same growth rate or maintenance costs at different light levels. The form of the model is also consistent with data from cultures of other phytoplankton (*Pavlova lutheri* and *Skeletonema costatum*; Sakshaug and Andersen, 1989; Chalup and Laws, 1990), as illustrated by model simulations in Supplementary Figure 2. Hence the model is not specific to a single organism, though each species requires different parameter values, representing inter-species variations in traits. We discuss the plausibility of inferred parameter values at the end of the Results section (see the subsection “Plausibility of the predicted macromolecular allocation”). We note that Equation (17) does not predict $\mu_{max}^{I}$, which requires additional constraints brought to bear through N and/or P limitation when the chlorophyll, C, N, and P conservation equations are coupled (see the subsection “What is the maximum growth rate for a given light intensity, $\mu_{max}^{I}$?”).

#### Why Does N:C Vary Linearly With Growth Rate, and Why Does It Change With Photon Flux?

Consider the case of nitrogen limitation, when allocation to nitrogen storage is small. In this case, to a first approximation, the cellular quota of nitrogen is dominated by that of protein (Liefer et al., 2019).

Nucleic acids account for <7% of cellular dry weight in phytoplankton (Parsons et al., 1984; Anderson, 1995), small relative to that in heterotrophic bacteria. Thus, for explanatory purposes we will use an approximate form of Equation (8) that considers only the contribution from protein (a full, un-approximated solution is provided in the Methods section and is used in the figures of model solutions). In the case where most nitrogen is associated with protein, Equation (8) becomes:


(18) $$\begin{array}{l} N:C\;\approx Y_{Pro}^{N:C}\left(Q_{C}^{Pro-Pho}+Q_{C}^{Pro-Bio}+Q_{C}^{Pro-Other}\right) \end{array}$$

Here nitrogen and carbon are linked by the constant elemental ratio for protein, $Y_{Pro}^{N:C}$ (Table 1). Other macromolecules have different elemental stoichiometries. $Q_{C}^{Pro-Other}$ represents the fixed, minimum complement of protein essential for the cell. The cellular investment in the photosynthetic protein is assumed to vary linearly with chlorophyll, and the investment in biosynthetic protein pools is assumed to vary linearly with growth rate using Equations (12, 14), respectively, as discussed above (in “Representing relationships between macromolecular pools and rates”). Combining Equations (12, 14, 17, 18) leads to an expression that describes the relationship between N:C of the population and growth rate under N-limiting conditions:


(19) $$\begin{array}{l} N:C\approx Y_{Pro}^{N:C}(\left(A_{Chl}\left(I\right)A_{Pho}+A_{Bio}\right)\mu \\ \;+\left(B_{Chl}\left(I\right)A_{Pho}+Q_{C}^{Pro-Other}\right)) \end{array}$$

**Figure 5:**
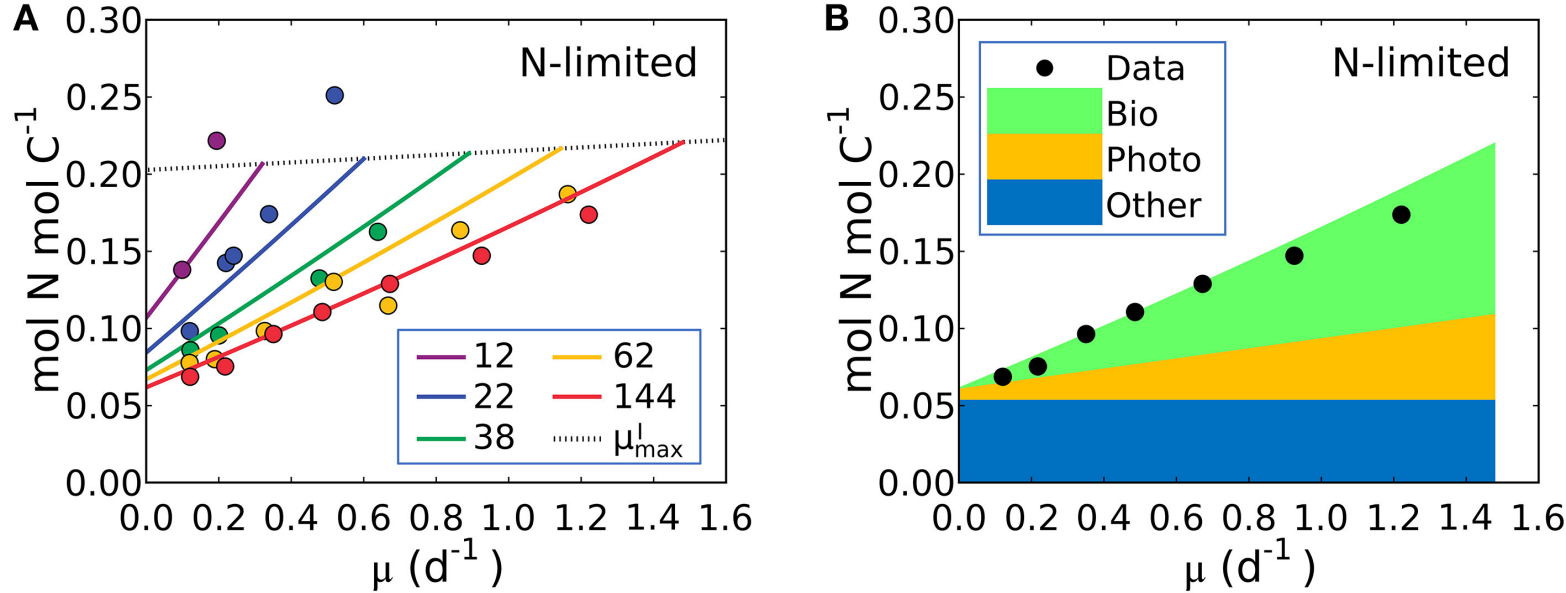
Model-data comparison of N:C and model prediction of macromolecular allocation of *Synechococcus linearis*. **(A)** N:C under N limitation under different light intensities; light intensities (μmol m^−2^ s^−1^) are in the legend. Curves are model results and points are data (Healey, 1985). Dotted lines represent $\mu_{max}^{I}$ at various light intensities; high $\mu_{max}^{I}$ for higher light intensity. **(B)** Macromolecular allocation in N, normalized by cellular C under the light intensity of 144 (μmol m^−2^ s^−1^). Black points are data for total values under the same light intensity (Healey, 1985). See the legend for color definitions: *Bio*, biosynthetic protein + RNA; *Photo*, chlorophyll + photosynthetic protein; *Other*, other molecules. *Bio* and *Photo* is dominated by biosynthetic protein and photosynthetic protein, respectively. Detailed macromolecule allocation in Supplementary Figure 3 and N:C under P limitation in Supplementary Figure 4.

Equation (19) predicts a linear relationship between N:C and growth rate which has decreasing slope and intercept as with photon flux, qualitatively consistent with the observed data in Figures 1D–F, 2B, 5A, Supplementary Figures 1, 2. Extending the model to include N storage allows predictions of N:C under P-limitation (Supplementary Figure 4) which is discussed later.

The model suggests that N:C increases with growth rate at a fixed light intensity because there is a linear increase in the investment in both biosynthetic protein (Equation 14) and photosynthetic protein, latter being the associated with the linear increase in Chl:C with growth rate at fixed light intensity (Equations 12, 17). Likewise, a reduction of light intensity at a fixed growth rate also demands a higher investment in both chlorophyll and photosynthetic proteins, hence the slope of N:C increases with decreasing light intensity.

Equation (19) can be fit to the data on *S. linearis* also using the Metropolis-Hastings procedure (see Methods). The values of parameters *m*, $v_{I}^{max},$ and *A*_*I*_ (and thus *A*_*Chl*_ and *B*_*Chl*_) were solved by fitting Equation (17) above so the N:C vs. μ data provide constraints on *A*_*Pho*_ and *A*_*Bio*_ (the parameters which scale photosynthetic protein to chlorophyll and biosynthetic protein to growth rate) as well as the fixed pool of “essential” protein, $Q_{C}^{Pro-Other}$. In Figure 5A we illustrate fitted solutions of the of the un-approximated version of Equation (19) (where the minor contribution to N:C from RNA and chlorophyll are also resolved). Similar simulations of *Pavlova lutheri* and *Skeletonema costatum* are shown in Supplementary Figure 2. The ability of the model to fit the data suggests that the model captures major processes.

The model result shows a similar relative increase in the investment in photosynthetic and biosynthetic protein at moderate to high light levels (Figure 5B, Supplementary Figures 2–4). At lower photon fluxes N:C increases more rapidly (Figure 5A) because there is a much higher demand for investment in photosynthetic machinery to achieve the same growth rate (Supplementary Figures 2–4). The model suggests that N:C increases linearly with growth rate at fixed light because investment in both biosynthetic and photosynthetic protein must increase linearly and protein investment dominates the N:C ratio. The un-approximated model (Equation 33 in Methods) suggests a non-linearity due to investment in RNA, but its overall contribution to the cellular nitrogen budget means that the non-linearity is very small and Equation (19) is a good approximation.

#### Why Does P:C Increase Non-linearly With Growth Rate?

A significant fraction of cellular phosphorus is present in nucleic acids, lipid membranes, and storage compounds such as polyphosphate. Consider the case for P-limited culture in which luxury storage is small and the cellular quota of phosphorus is approximated by the sum of the three pools:


(20) $$\begin{array}{l} P:C\approx Q_{P}^{Thy}+Q_{P}^{RNA}+Q_{P}^{Other} \end{array}$$

where $Q_{P}^{Other}$ groups relatively stable pools of P: $Q_{P}^{Other}=Q_{P}^{DNA}+Q_{P}^{Other0}$, required even in the absence of net growth.

Here we invoke two of the fundamental relationships discussed earlier: the investment in thylakoid phospholipid, $Q_{P}^{Thy}$, is assumed linearly proportional to chlorophyll (Equation 13) and the investment in RNA is modeled as linearly proportional to total protein content and growth rate (Equation 15). (These models, and the evidence for them, are discussed in the section entitled “*Representing relationships between macromolecular pools and rates*”). Substituting Equations (13, 15, 3, 12, 14, 17) (in this order) into Equation (20), yielding a quadratic relationship between cellular P:C with growth rate:


(21) $$\begin{array}{l} P:C\;\approx \;a\mu^{2}+b\mu +c \end{array}$$

where


$$\begin{array}{l} a=A_{RNA}^{P}A_{Pho}A_{Chl}\left(I\right)+A_{RNA}^{P}A_{Bio}\\ b=A_{Pho}^{P:Chl}A_{Chl}\left(I\right)+A_{Pho}A_{RNA}^{P}B_{Chl}\left(I\right)+A_{RNA}^{P}Q_{C}^{Pro-Other}\\ c=A_{Pho}^{P:Chl}B_{Chl}\left(I\right)+Q_{P,min}^{RNA}+Q_{P}^{Other} \end{array}$$

**Figure 6:**
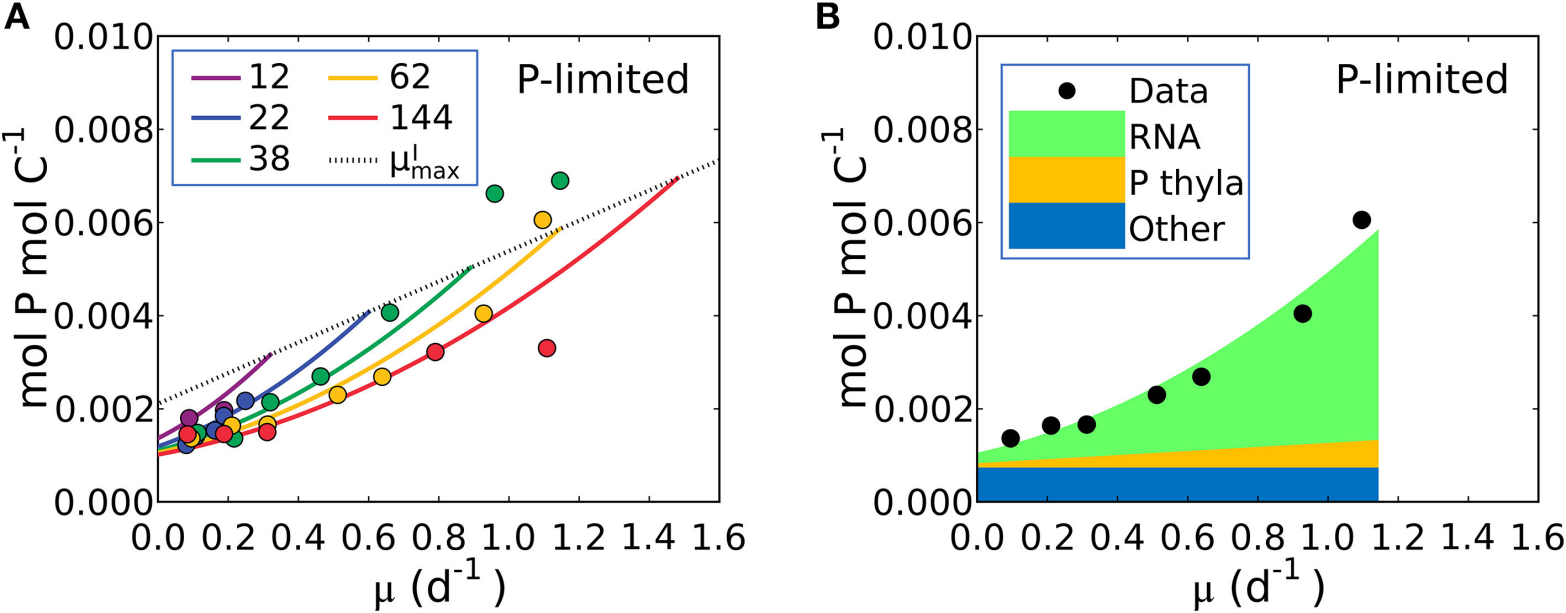
Model-data comparison of P:C and model prediction of macromolecular allocation in P for *Synechococcus linearis*. **(A)** P:C under P limitation under different light intensities; light intensities (μmol m^−2^ s^−1^) are in the legend. Curves are model results and points are data (Healey, 1985). Dotted line represents P:C at $\mu_{max}^{I}$ for various light intensities; high $\mu_{max}^{I}$ for higher light intensity. **(B)** Macromolecular allocation in P, normalized by cellular C under the light intensity of 62 (μmol m^−2^ s^−1^). Black points are data for total values under the same light intensity (Healey, 1985). See the legend for color definitions: *P thyla*, P in thylakoid membranes; *Other*: other molecules. Detailed macromolecule allocation in Supplementary Figure 3 and P:C under N limitation in Supplementary Figure 4.

Equation (21) predicts a quadratic relationship of P:C with growth rate, μ, is qualitatively consistent with the non-linear relationship in the P-limited cultures of *S. linearis* (Figure 1H), marine *Synechococcus* (WH8102) and *Selenastrum minutum* (Figure 1H). The qualitative fit enabled the optimization of parameters to match the *S. linearis* data (using the Metropolis Hastings algorithm, see Methods) and the resulting fit is shown in Figure 6A. Using the optimized parameters, the model provides a prediction of the allocation of phosphorus, shown as a function of growth rate at a single light intensity in Figure 6B. $Q_{P}^{Thy}$ increases linearly with growth rate for a fixed photon flux (Figure 6B) in concert with $Q_{C}^{Chl}$ and the photosynthetic proteins (Figures 4, 5A). The inference is that the non-linear relationship of P:C with growth rate (Figure 6B) is due to the investment in phosphorus-rich RNA (Figure 6B) which increases in proportion to both the growth rate and the cellular quota of protein, which also increases with growth rate due to investment in biosynthesis and light harvesting (Figure 5B). A similar non-linear relationship of P:C vs. μ was shown to be consistent in the culture of *Selenastrum minutum* (Elrifi and Turpin, 1985; Ågren, 2004).

#### What Is the Maximum Growth Rate for a Given Light Intensity, $\mu_{max}^{I}$?

Expanding the cellular carbon quota in terms of the macromolecular components as described by Equations (2)–(6), and accounting for only the most quantitatively influential molecules in order to provide an analytic solution (the full, un-approximated model is described in Methods), we describe carbon allocation in the cell as


(22) $$1\approx Q_{C}^{Pro}+Q_{C}^{Carb-Other}+Q_{C}^{Lip-Other}+Q_{C}^{Carb-Sto}+Q_{C}^{Lip-Sto}$$

Using Equation (3), we can further resolve the proteomic contributions into photosynthetic, biosynthetic, structural/other:


(23) $$\begin{array}{l} 1\approx Q_{C}^{Pro-Pho}+Q_{C}^{Pro-Bio}+Q_{C}^{Other}+Q_{C}^{Csto} \end{array}$$

where $Q_{C}^{Other}\approx Q_{C}^{Pro-Other}+Q_{C}^{Carb-Other}+Q_{C}^{Lip-Other}$ and $Q_{C}^{Csto}=\;Q_{C}^{Carb-Sto}+\;Q_{C}^{Lip-Sto}$. Using Equations (12, 14, 17), we can solve Equation (23) for the population growth rate, μ;


(24) $$\begin{array}{l} \mu \approx \frac{\left(1-Q_{C}^{Other}-Q_{C}^{Csto}\right)v_{I}\left(I\right)-A_{Pho}m}{A_{Bio}v_{I}\left(I\right)+A_{Pho}\left(1+E\right)} \end{array}$$

This equation indicates that as the investment in carbon storage $Q_{C}^{Sto}$ decreases, growth rate μ increases.

**Figure 7:**
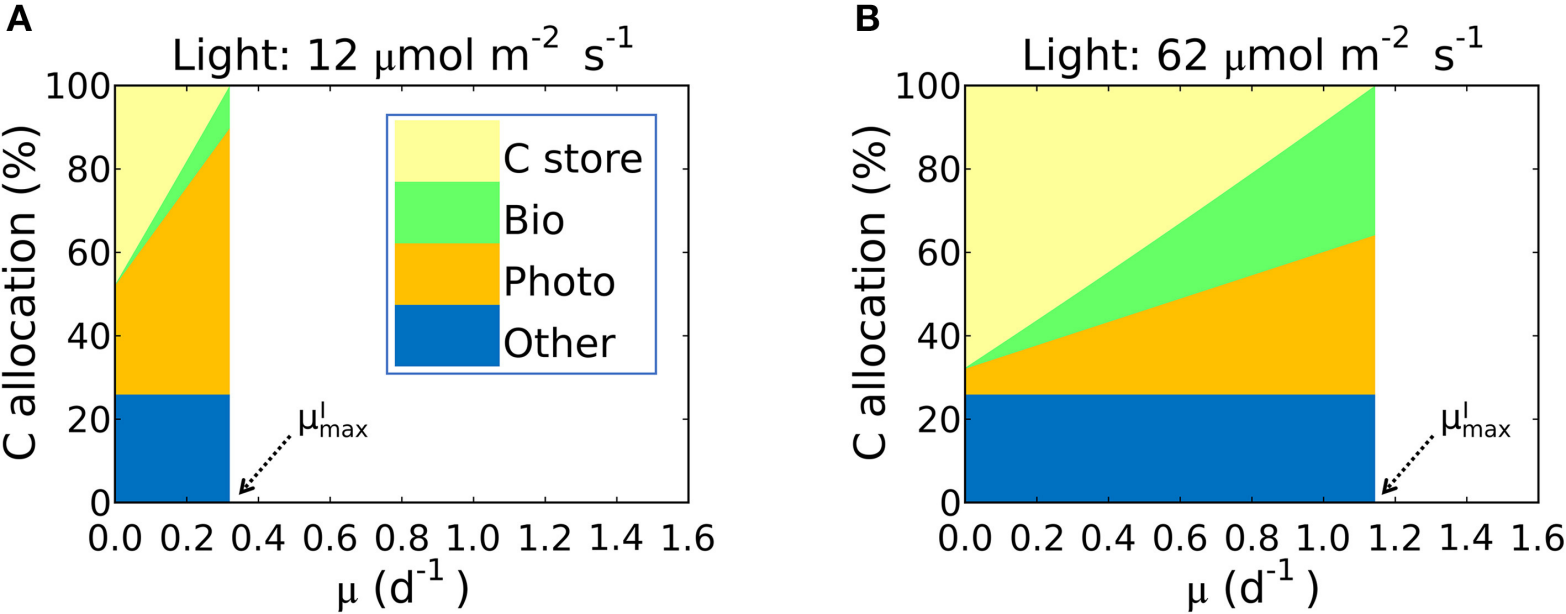
Simulated carbon allocation and $\mu_{max}^{I}$ of *Synechococcus linearis* under N limitation. The light intensities are **(A)** 12 and **(B)** 62 (μmol m^−2^ s^−1^). See **(A)** for color definitions: *C store*, C storage; *Bio*, Biosynthetic protein + RNA; *Photo*, Chlorophyll + Photosynthetic protein + P-lipid in thylakoid membranes; *Other*, other molecules with constant cellular investment. *Bio* and *Photo* are dominated by biosynthetic and photosynthetic proteins, respectively. C allocation and $\mu_{max}^{I}$ of *Synechococcus linearis* under P limitation in Supplementary Figure 5. C allocations of other species (*Pavlova lutheri* and *Skeletonema costatum*) in Supplementary Figure 6.

This inference is logical: the maximum growth rate for a given light intensity, $\mu_{max}^{I}$, should occur when as much biomass can be allocated to growth related macromolecules as possible; in other words when carbon storage is minimal and $Q_{C}^{Csto}$ approaches 0. In Figure 7 we illustrate this in terms of carbon allocation as a function of growth rate in model solutions where the parameters were fitted for *S. linearis*. Solutions are shown for two light levels: at low light, the rapidly increasing allocation to photosynthetic machinery as a function of growth rate means that cellular allocation to storage becomes small (and allocation to functional machinery becomes large) at quite a low growth rate. In contrast, at high light, the lower demand for photosynthetic apparatus allows a greater investment in biosynthesis and higher maximum growth rate (Figure 7, Supplementary Figures 5A, 6).

**Figure 8:**
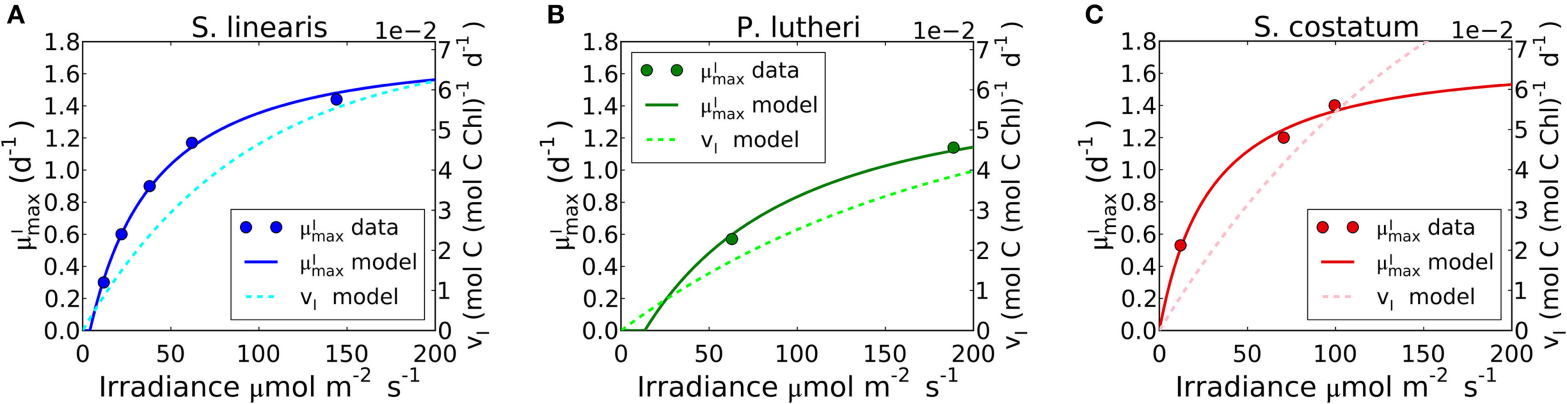
Simulated light dependence of maximum growth rate ($\mu_{max}^{I}$) and photosynthesis rate (*v*_*I*_) under N limitation for three different phytoplankton. **(A)** *Synechococcus linearis*, **(B)** *Pavlova lutheri*, and **(C)** *Skeletonema costatum*. Modeled $\mu_{max}^{I}$ is compared to data (Healey, 1985; Sakshaug and Andersen, 1989; Chalup and Laws, 1990). There is one data point at the light intensity of 1,203 (μmol m^−2^ s^−1^) for *Skeletonema costatum* not included or considered since the cells were likely photodamaged under such high light intensity, negatively altering $\mu_{max}^{I}$. Light dependence of $\mu_{max}^{I}$ of *Synechococcus linearis* under P limitation in Supplementary Figure 5, where $\mu_{max}^{I}$ is almost identical as **(A)**, similarly reproducing the data (Healey, 1985).

The limit of the model, which occurs when $Q_{C}^{Csto}$ approaches zero in Equation (24), reproduces the observed $\mu_{max}^{I}$–light relation of *S. linearis* and two marine phytoplankton (*Pavlova lutheri* and *Skeletonema costatum*; Figure 8, Supplementary Figure 5B). The $\mu_{max}^{I}$ curve ultimately saturates because photosynthesis per chlorophyll *v*_*I*_ saturates. When $\mu_{max}^{I}$ increases, due to an increase in light, there is a decreased investment in light-harvesting proteins which is traded off against increased biosynthetic proteins and a higher maximum growth rate. This is qualitatively consistent with recent proteomic studies (McKew et al., 2013; Jahn et al., 2018; Zavřel et al., 2019).

#### Differences Between $\mu_{max}^{I}$ and *v*_*I*_

Growth rates and photosynthesis are often used interchangeably and the relationships for photosynthesis and light have often been applied to growth rates in ecosystem models (Moore et al., 2004; Buitenhuis et al., 2013; Dutkiewicz et al., 2015; Coles et al., 2017). However, photosynthesis and growth (biosynthesis) are metabolically distinct and need not be equivalent. The two rates are qualitatively similar because both share a saturating dependence on light (Figure 8)_._ Growth rate has a non-zero intercept on the light axis, which represents the minimum light intensity required for cellular maintenance. Notably, $\mu_{max}^{I}$ approaches the saturated value at a lower light intensity than *v*_*I*_. This can be seen in Figure 8 where the model is fit to data sets for *S. linearis* (Healey, 1985) and two photosynthetic algae (Sakshaug and Andersen, 1989; Chalup and Laws, 1990). As photon flux decreases, investment in photosynthetic apparatus increases rapidly (Figure 7, Supplementary Figures 5A, 6) at the expense of biosynthetic machinery and such that $\mu_{max}^{I}$ saturates at a lower light intensity than *v*_*I*_. This highlights the high cost of the photosynthetic apparatus.

#### What Does N:P Depend Upon?

The N:P ratio of plankton has been a topic of interest going back to Redfield (1934, 1958). Could the framework presented above be used to predict and interpret the N:P of primary producers? The answer is not immediately clear: Equations (19, 21) represent the N:C ratio under N limitation and P:C under P limitation. Simply dividing the two does not provide an accurate prediction of N:P because, typically, one resource is limiting and the other in excess in the environment, and non-limiting resources accumulate in intracellular storage pools.

**Figure 9:**
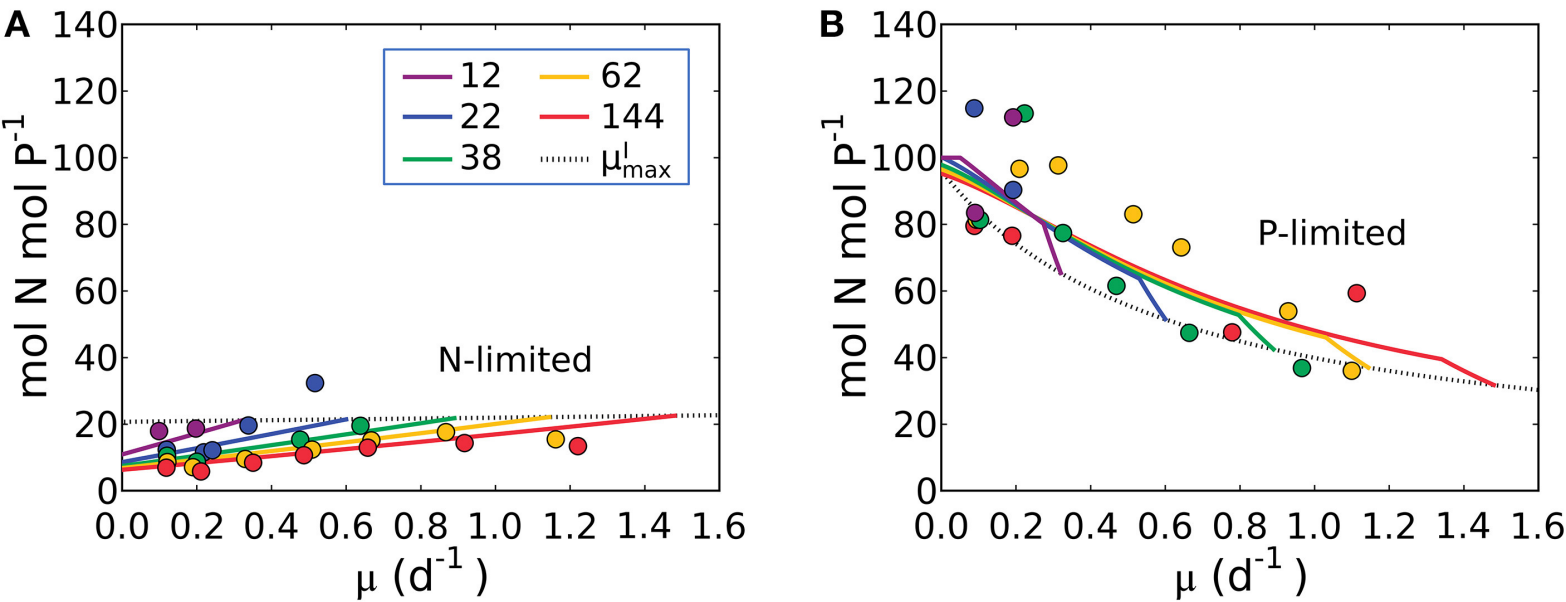
Model-data comparison of N:P. **(A)** Under N limitation. **(B)** Under P limitation. Curves: model. Points: data of *Synechococcus linearis* (Healey, 1985). Dotted black line indicates values at $\mu_{max}^{I}$ for various light intensities. Numbers in the legend show light intensities (μmol m^−2^ s^−1^).

Using the comprehensive data set of Healey (1985), it is possible to quantify the storage of N and P when each is the non-limiting resource. For stoichiometric purposes, we assume that P-storage is in the form of polyphosphate which has no carbon content and that N-storage is in the form of cyanophycin (Table 1). The storage capacity of the cells cannot be predicted a priori so the model allows cells to take up and store the non-limiting resource (N or P) until a maximum storage is reached, constrained by the observed elemental stoichiometry. Thus, we introduce two new, empirically constrained parameters; the maximum storage capacities for N and P (see the subsection “Evaluating cellular C concentration and N and P storage” in Methods for details). Using this approach, we can model N:P under both N and P limitation (illustrated in Figure 9), as well as N:C under P limitation and P:C under N limitation (Supplementary Figure 4). In Figure 9, model parameter values are constrained (as discussed above) with data for *S. linearis*, including the maximum storage capacities.

The model qualitatively captures the variations in N:P with growth rate under both N and P limitation. Under N-limitation, N:P (Figure 9A) has a linearly increasing trend with growth rate, following N:C (Figure 5) because of the increasing investment in N-rich proteins with growth rate. In this case P:C is relatively constant regulated by the contribution of P storage (Supplementary Figure 4B). Under P-limitation, N:P declines rapidly with growth rate (Figure 9B) because the increase in RNA with growth rate is quadratic (Figure 6), while that of protein is linear (Supplementary Figure 4A), so the investment in P increases more rapidly. N storage is a relatively moderate contribution to the cellular N quota (Supplementary Figure 4A). The model with the N and P storages captures and interprets the trends of N:P observed in the laboratory study by simply allowing storage up to an empirically informed limit. Further basic study of the dynamics of, and limits to, nutrient storage pools would be necessary to inform a model rooted in first principles.

#### Plausibility of the Predicted Macromolecular Allocation

**Figure 10:**
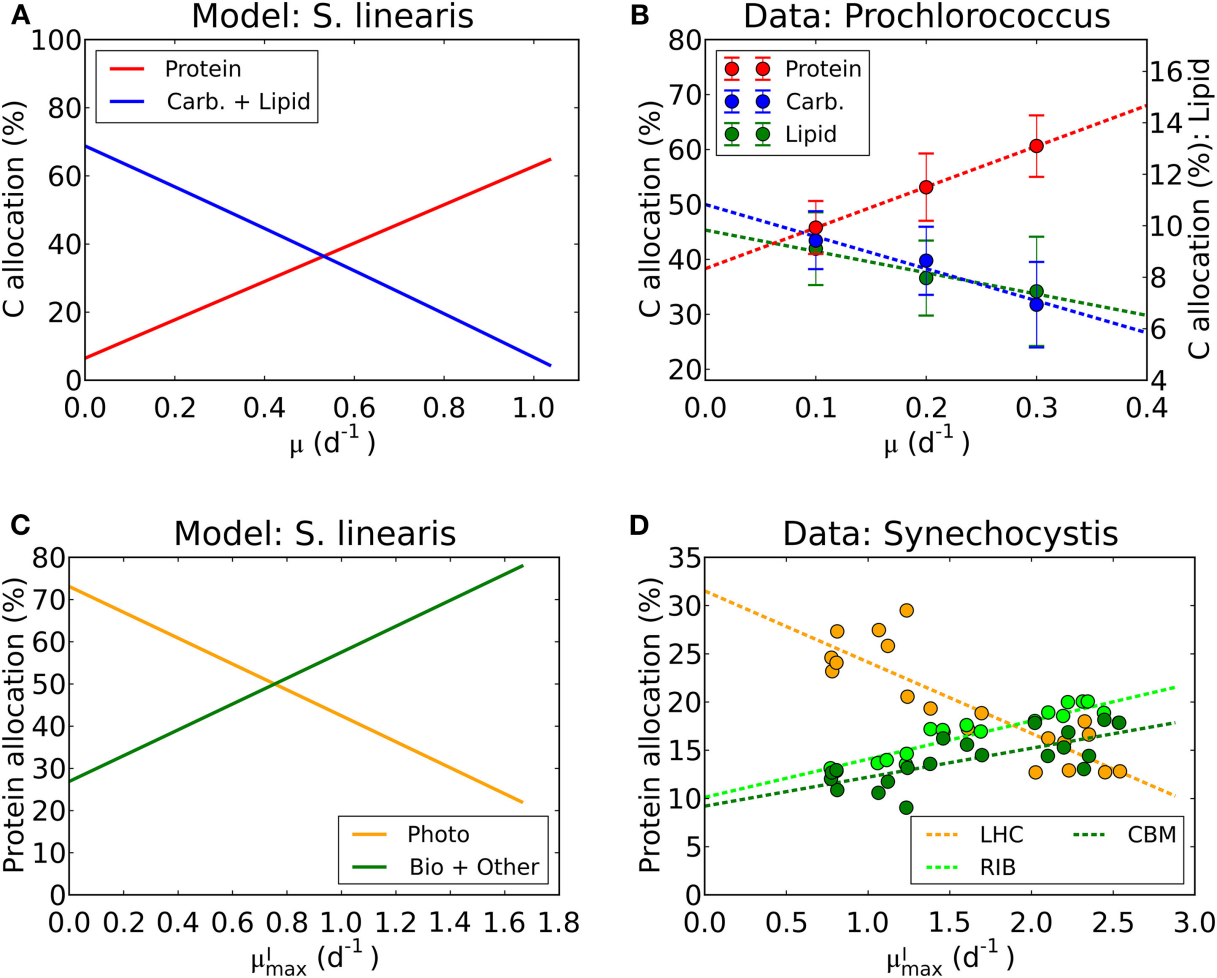
Model-data comparison of macromolecular allocation. **(A)** Modeled C allocation of *Synechococcus linearis* at the light intensity of 50 μmol m^−2^ s^−1^ under N limitation. *Carb*.: carbohydrate. **(B)** Measured C allocation of *Prochlorococcus marinus* (PCC 9511) at the light intensity of ~50 μmol m^−2^ s^−1^ under N limitation (Felcmanová et al., 2017). C allocation (%) for protein and carbohydrate (*Carb*.) are on the left axis and that for lipid is on the right axis. Dotted lines are linear interpolation and error bars are the standard deviation. **(C)** Modeled protein allocation of *Synechococcus linearis* at $\mu_{max}^{I}$, nutrient replete growth rate. Here changes in $\mu_{max}^{I}$ are caused by changes in light intensities. *Photo*, photosynthetic proteins; *Bio*, biosynthetic proteins; *Other*, other proteins. **(D)** Observed protein allocation in *Synechocystis* sp. (PCC 6803) (Jahn et al., 2018). *LHC*, light-harvesting complex; *RIB*, ribosome and protein production; *CBM*, proteins for C uptake, fixation and metabolism. Compare the general trends in *Photo* with *LHC* and *Bio* + *Other* with *RIB* and *CBM*.

We have used laboratory data on elemental stoichiometry to constrain a model which resolves macromolecular allocation. As such, the model makes testable predictions. Our estimated photosynthetic parameters ($v_{I}^{max}$ and *A*_*I*_) sit within the range of observation (Platt et al., 1980; Cullen, 1990; Moore and Chisholm, 1999) for the given range of chlorophyll vs. μ (Figure 4, Supplementary Figure 2). While there are not direct macromolecular or proteomic data available for the particular laboratory studies which we simulated, some recent culture studies have resolved macromolecular and proteomic allocation. The predictions of changes in macromolecular allocation (e.g., Proteins, Lipids+ Carbohydrate and RNA) with growth rate and light are qualitatively similar to those observed in a recent laboratory studies (Liefer et al., 2019). Also, in Figures 10A,B, we compare the inferred allocation to protein, carbohydrates and lipids as a function of growth rate for *S. linearis* with measurements of these bulk macromolecular pools in chemostat cultures of *Prochlorococcus marinus* (PCC 9511) (Felcmanová et al., 2017). The model qualitatively captures the observed trends in allocation and the general magnitude of the observed pools, though the specific values differ due to either inter-species differences or model limitations. In Figures 10C,D, we also compare the predictions for broad-scale protein allocation with light-dependent growth rate (i.e., maximum growth rate for a given light intensity) from the constrained *Synechococcus* model, and direct observations of similar proteomic categories from turbidostat cultures of *Synechocystis* (Jahn et al., 2018). Again, there is a qualitative agreement between the predicted trends: as the light-limited growth rate increases, the investment in light-harvesting proteins declines while the investment in biosynthesis increases. The inferred allocation to photosynthetic proteins tends to be rather high relative to the direct proteomic study, likely reflecting inter-species differences. Direct laboratory studies where elemental stoichiometry, macromolecular composition, and proteomics are all concurrently measured are possible and would allow a more strenuous test and calibration of such models.

## Discussion

### A Model of the Elemental Stoichiometry of Phytoplankton in Relation to Light Intensity, Growth Rate, and Limiting Resource

**Figure 11:**
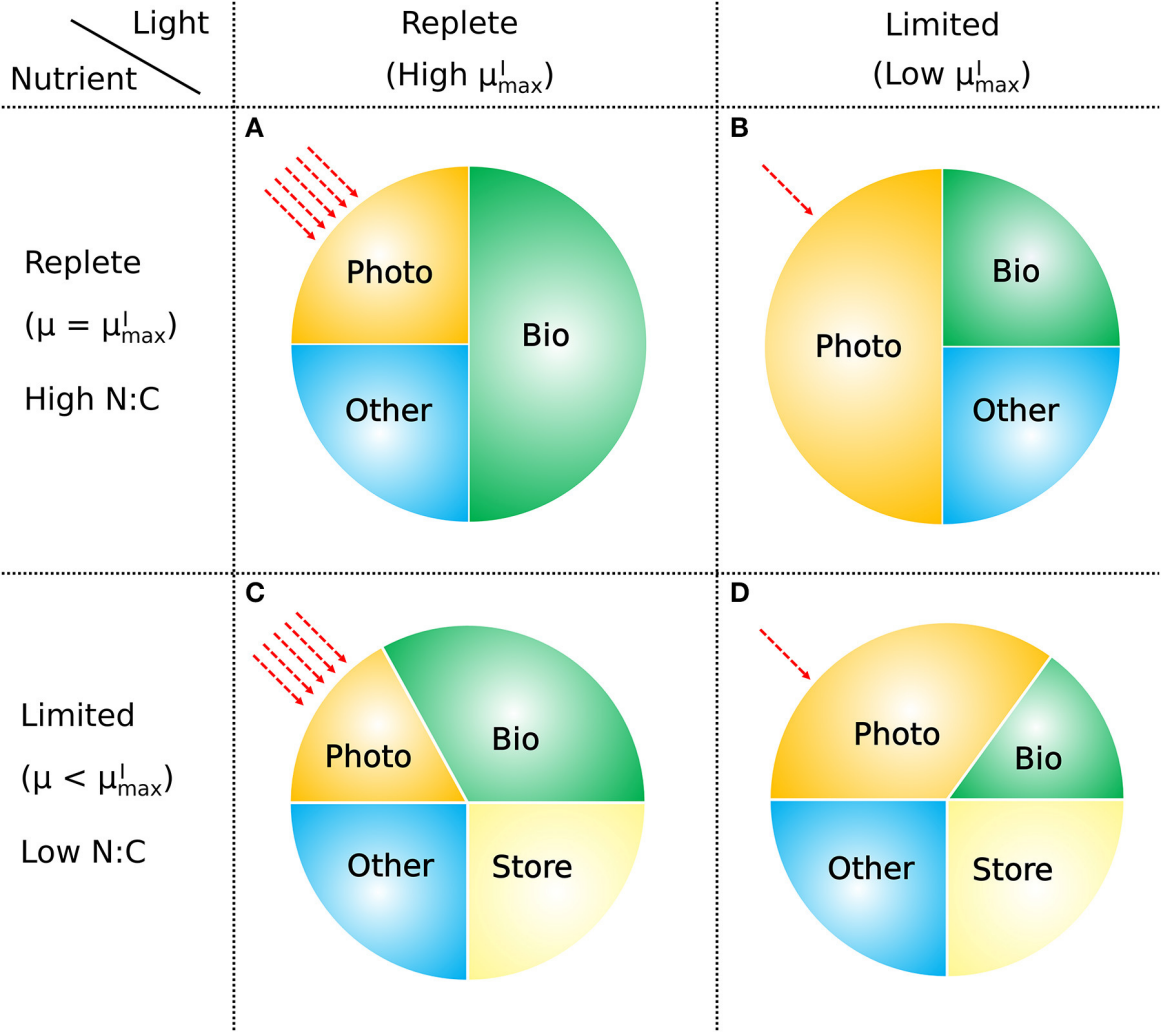
How macromolecular allocation of phytoplankton responds to light and nutrient, constraining the growth rate based on CFM-Phyto. *Bio*, biosynthetic molecules; *Photo*, photosynthetic molecules; *Other*, other constant molecules; *Store*, storage molecules. Red dashed arrows represent light intensities. Here the growth rate μ is proportional to the ratio of *Bio*. When light and nutrient are sufficient, there is a high ratio of *Bio* with high $\mu_{max}^{I}$ (the maximum viable growth rate at a certain light intensity) **(A)**. Light limitation increases the fraction of *Photo* limiting that of *Bio* leading to lower $\mu_{max}^{I}$ **(B)**. Nutrient limitation leads to a smaller allocation to photosynthetic and growth-related molecules (*Photo, Bio*) leading to a growth rate μ lower than $\mu_{max}^{I}$ **(C)**. Under both light and nutrient limitation, these effects are combined, further limiting the ratio of *Bio*, thus limiting the growth rate **(D)**. C storage lowers N:C of phytoplankton.

The model presented above provides a conceptually simple, yet quantitative description of the relationship between the elemental stoichiometry of phytoplankton, their growth rate, resource availability, and macromolecular allocation (Figure 11). It is based on a straightforward accounting of the allocation between and within major pools of macromolecules, along with four representations of the relationships between pools and fluxes. These are (i) a saturating relationship between light intensity and photosynthetic efficiency, (ii) a constant ratio of chlorophyll to other light harvesting and photosynthesis apparatus, (iii) a linear relationship between allocation of biosynthetic protein and growth rate, and (iv) a linear relationship between RNA:protein and growth rate. Representations (i), (iii), and (iv) are empirically driven, while (ii) is hypothetical, though simple and logical. The ability of the model to fit laboratory data for diverse phytoplankton taxa indicates that the model framework is generally applicable.

The framework of the model is conceptually simple and steady-state solutions can be solved algebraically and parameters optimized to match empirical data. We have used it to model and interpret laboratory data relating the elemental stoichiometry of *S. linearis* to growth rate and light intensity under both N- and P-limiting conditions. The stoichiometric data provide indirect constraint on macromolecular allocation, which imply a common set of allocation strategies amongst phytoplankton. Below, we discuss some of the limitations and simplifications of the approach.

By explicitly resolving the macromolecular allocation, the model captures and provides a simple interpretation for the contrast between cellular nitrogen quota (or N:C ratio) which varies linearly with growth rate under N-limitation, and the phosphorus quota (or P:C) which varies non-linearly with growth rate under P-limitation. At fixed light intensity and under nitrogen limitation, cellular protein increases linearly with growth rate (Felcmanová et al., 2017), driving the linear trend in N:C vs. μ captured in Equation (19). In contrasts, the observed linear relationship between RNA:protein (Nicklisch and Steinberg, 2009) combines with the increase in protein with growth rate (Rhee et al., 1981; Liefer et al., 2019) to drive the non-linear relationship of P:C with growth rate, captured in Equation (21). This contrast was also captured in the more abstract model of Ågren (2004). The explicit macromolecular resolution also allows the model to capture the observed decrease of N:P with growth rate under P limitation (Perry, 1976; Elrifi and Turpin, 1985; Healey, 1985; Garcia et al., 2016; Figure 9B) which is driven by the increasing RNA/protein ratio with μ (Bremer and Dennis, 1996; Nicklisch and Steinberg, 2009; Scott et al., 2010).

We suggest that the explicit resolution of macromolecular reservoirs provides an important advantage over more idealized frameworks. It allows the exploitation of key observed relationships (e.g., RNA:protein vs. μ) and it explicitly couples the dynamics of N, P and C providing a comprehensive framework (c.f. N:C only in the case of Geider et al., 1998, for example). The more idealized internal-stores model (Droop, 1968) treats all resources alike and implicitly does not capture this contrast in N:C and P:C variations with μ, unless significantly different parameters are applied between N:C and P:C. The chain model (Pahlow and Oschlies, 2009) differentiates the dynamics of N and P, but still remains abstracted from the macromolecular foundations. It predicts non-linear relationships for both N:C and P:C with μ, which also leads to compensation and predictions of a rather constant N:P under P limitation (Pahlow and Oschlies, 2009), whereas as the data generally shows decreasing N:P with μ (Perry, 1976; Elrifi and Turpin, 1985; Healey, 1985; Garcia et al., 2016) as our model predicts (Figure 9B).

We suggest that the explicit macromolecular representation also has some interpretive and predictive advantages. It has the potential to be directly compared with direct proteomic and macromolecular observations (McKew et al., 2013; Felcmanová et al., 2017; Jahn et al., 2018; Liefer et al., 2019; Zavřel et al., 2019), which will leverage new data sets and technologies more directly. For example, the calibrated model for *Synechoccocus linearis* presented above indicates a strong relationship between investment in photosystem proteins and light intensity. Under fixed light intensity, both biosynthetic and photosynthetic proteins increase with growth rate. However, under light co-limitation, when varying the growth rate (i.e., $\mu_{max}^{I}$), by changing light intensity, the model predicts a reduced requirement for photo-proteins with increasing light accompanied by an increase in biosynthetic protein, consistent with data from proteomic studies (McKew et al., 2013; Jahn et al., 2018; Zavřel et al., 2019). This mechanism stabilizes the amount of total protein and explains relatively stable N:C with simultaneously varied light and growth rate (Geider et al., 1985).

The model illustrates the relationship between the maximum growth rate at a given light intensity and storage. In order to increase the growth rate, cells invest in protein at the expense of storage compounds. The maximum growth rate for a given light intensity occurs when storage is minimized and functional allocation is maximized. In some circumstances, maximizing growth rate will be the best measure of fitness, but in others storage is likely to be advantageous. For example, if we consider phytoplankton-bloom conditions, maximizing growth rate may be more important since in such situations, phytoplankton with faster growth can outcompete others and dominate the region (Dutkiewicz et al., 2009). However, in environments where the nutrient level rapidly fluctuates (e.g., with time scale of days), phytoplankton with high storage capacity might be advantageous by being able to grow under nutrient depletion with stored nutrients before another pulse of high nutrient occurs (Tozzi et al., 2004; Grover, 2009, 2011).

### Generality of the Model

While we have focused our development and discussion around the data set of Healey (1985) for *S. linearis*, the framework is sufficiently coarse-grained and rooted in basic, common physiology that it is qualitatively compatible with data from numerous phytoplankton, spanning a wide range of cell size and taxonomic groups (Figure 1). We have fit the same framework to several of these data sets (e.g., Figures 4–6, 8, Supplementary Figure 2; Healey, 1985; Sakshaug and Andersen, 1989; Chalup and Laws, 1990). While different parameter values are required, reflecting different allocation strategies or traits, the basic framework is general, predicting the common trends from the laboratory studies (Figures 1, 2, Supplementary Figure 1). However, the allocation strategies (and parameter values) differ between species. The use of different nitrogen substrates (e.g., nitrate vs. ammonium) could be represented by changes to the respiratory cost of synthesis, *E* (Rittmann and McCarty, 2001).

### Model Simplifications and Limitations

The model presented here represents an attempt to provide a minimal, transparent and biologically meaningful framework which relates allocation between and within the major macromolecular pools to elemental ratios and growth rates under diverse environmental conditions. It is framed so that the internal allocation is, in principle, in terms of measurable quantities (though not all were available in the data sets studied here). These measurable pools can be mapped into categories which are not directly measurable, but which are grouped by function (Figures 3B, 11), in the spirit of allocation models (Shuter, 1979; Scott et al., 2010). This functional mapping enables simple interpretations of the relationships of interest.

As with any quantitative model, there is a trade-off between realism, data constraints and insight, and we have not resolved a number of potentially important factors. We have assumed a fixed composition of thylakoid membranes which may vary in reality. In particular, the fraction of light harvesting machinery might change relative to other components, which would alter the chlorophyll to protein or lipid ratio. The model could be improved with an additional layer of detail, separating the light harvesting and other components. To constrain the model, combined measurements of chlorophyll and proteomics (e.g., McKew et al., 2013, 2015; Zavřel et al., 2019) as well as thylakoid lipid would be useful. Photo-inhibition has not been addressed here and presumably would demand the resolution of photoprotective proteins (Geider et al., 2009). Healey's (1985) data set focused on lower photon fluxes so this case was not addressed here. We have also constrained a single set of photosynthesis-vs.-light parameters to simulate experiments at all light levels, though we are aware that acclimation would likely modify them, however we found no significant improvement in model fits when allowing this extra degree of freedom. We have not addressed the potential for variations in allocation and elemental ratios as a function of temperature, though it is known that there are indeed sensitivities (Thrane et al., 2017) and this could be an interesting and important extension to the study. We have also not explicitly resolved allocation to nutrient uptake transporters, which varies with nutrient concentration (McKew et al., 2015; Lin et al., 2016). However, in overall elemental stoichiometry, the influence appears limited since proteomic studies suggest that allocation to the nutrient transporter proteins is modest (e.g., on the order of 10^−1^% of total spectral counts in a recent proteomic study; McKew et al., 2015) relative to investments in light harvesting and biosynthesis (McKew et al., 2013; Jahn et al., 2018; Zavřel et al., 2019). Also, investment in transporters increases under low nutrients (McKew et al., 2015), so if the transporter is a dominant part of protein, it would lead to high N:C at low growth rate, but the data show otherwise (Figure 1, Supplementary Figure 1). Hence, we suggest that investment in transporters is a next-order effect which would have a small impact for elemental stoichiometry in the phytoplankton addressed here. Resolution of transporter allocation would also introduce further unconstrained parameters. Other studies have placed more emphasis on the allocation to transporters showing it to be an important factor in expressing phenotypic diversity and acclimation to different nutrient and light regimes (Smith et al., 2009, 2016; Bonachela et al., 2013; Garcia et al., 2016; Chen et al., 2019).

We have also neglected the substitution of non-P-lipids for P-lipids under low P concentrations (Van Mooy et al., 2009), which has a significant impact on the P budget of the cell, and focused on the major macro-nutrient elements (C, N, P) though trace metal allocation is also of significance (Ho et al., 2003; Saito et al., 2011). The allocation of trace metals to specific protein groups would provide a way to link them in such a model. As discussed above, storage of non-limiting elements is important for consideration of the N:P ratio and the applicability of the Growth Rate Hypothesis (specifically under N-limitation). The limits to storage and maximum quotas are not clearly defined at present and worthy of further work. Such simplifications and omissions could ultimately be addressed with coordinated laboratory and modeling studies.

### Perspective and Outlook

Despite the limitations of the study listed above, we have shown that a conceptually simple model rooted in mass balance and a few basic, empirically sound representations can capture the relationships between growth rate and elemental stoichiometry under a variety of environmental conditions accurately. We suggest that the explicit representation of measurable macromolecular pools allows an advantage over more abstracted forms rooted in elemental quotas. It allows the exploitation of key physiological observations such as the changes in RNA:protein with μ, as well as testable predictions regarding macromolecular allocation. Parameters controlling rates and allocation can be calibrated with laboratory data, either inverted from stoichiometric data as we have done here, or directly measured (McKew et al., 2013, 2015; Felcmanová et al., 2017; Jahn et al., 2018; Zavřel et al., 2019) though this is not yet routinely the case.

Physiological models of “intermediate complexity” such as this have a role to play in ecological and biogeochemical studies. While Monod (1949) and Droop (1968) kinetics provide much simpler frameworks which have fewer parameters and are mathematically convenient, they lack some important biological detail, especially if one wishes to relate elemental stoichiometry to growth rate and environment. The approach presented here, while still idealized, is economical and, with some modifications, could be efficiently employed in biogeochemical and ecological simulations. While modern Flux Balance Analysis approaches now allow genome-scale representations of microbial physiology (Orth et al., 2010) they are typically subject to an imposed macromolecular composition (the “biomass function”) which is generally empirically determined and invariant and so do not address the elemental stoichiometry of the cell prognostically. Laboratory studies reveal visible changes in biomass function (i.e., the relative allocation to different macromolecules) over reasonable ranges of environmental conditions (Rhee, 1978; McKew et al., 2013, 2015; Felcmanová et al., 2017; Jahn et al., 2018; Liefer et al., 2019; Zavřel et al., 2019). Thus, models of the type presented here complement, and could potentially couple to, more detailed genome-scale simulations. We suggest that integrated laboratory and modeling studies in which a comprehensive set of physiological measurements (i.e., elemental stoichiometry, proteome, transcriptome) and a hierarchy of models (coarse-grained and genome-scale) would be valuable.

## Methods

### Full Model Description and Parameter Estimation

Here we provide a complete version of the model: CFM-Phyto (Figure 3). We first detail the organization of macromolecular components into four functional classes (*Photo, Bio, Store*, and *Other*). Then we discuss how maximum growth rate, $\mu_{max}^{I}$, can be predicted from macromolecular allocation. Then we provide details on how storage, population density, elemental stoichiometry and carbon biomass density are evaluated. Finally, we describe how model parameters are estimated.

### Re-framing the Model According to Functional Allocation

Equations (2)–(6) lead to the following accounting for total cellular carbon in various macromolecular pools:


(25) $$\begin{array}{l} 1=Q_{C}^{Pro-Pho}+Q_{C}^{Pro-Bio}+Q_{C}^{Pro-Other}+Q_{C}^{RNA}+Q_{C}^{DNA}+Q_{C}^{Chl}\\ \;+Q_{C}^{Plip-Thy}+Q_{C}^{Nsto}+Q_{C}^{Csto}+Q_{C}^{Other0} \end{array}$$

where $Q_{C}^{Other0}=Q_{C}^{Carb-Other}+Q_{C}^{Lip-Other}$. The cellular pools, defined in the main text, are described in carbon units, relative to total cellular carbon (mol C mol^−1^ C). The cellular components can be re-arranged and gathered into four functional classes, as depicted in Figure 3B:


(26) $$\begin{array}{l} 1=Q_{C}^{Pho}+Q_{C}^{Bio}+Q_{C}^{Sto}+Q_{C}^{Other} \end{array}$$

where


$$\begin{array}{l} Q_{C}^{Pho}=Q_{C}^{Pro-Pho}+Q_{C}^{Chl}+Q_{C}^{Plip-Thy}\\ Q_{C}^{Bio}=Q_{C}^{Pro-Bio}+Q_{C}^{RNA}\\ Q_{C}^{Sto}=Q_{C}^{Nsto}+Q_{C}^{Csto}\\ Q_{C}^{Other}=Q_{C}^{Pro-Other}+Q_{C}^{DNA}+Q_{C}^{Other0} \end{array}$$

$Q_{C}^{Pho}$ includes all components in the thylakoid membranes, which are allocated according to the light intensity and growth rate. The model assumes that all components of $Q_{C}^{Pho}$ are adjusted in concert; i.e., the relative proportions of the components of $Q_{C}^{Pho}$ are fixed and independent of variations in the magnitude of $Q_{C}^{Pho}$. In other words, the makeup of the photosynthetic machinery is invariant. $Q_{C}^{Bio}$ contains all components whose allocation depends mostly on growth rate. $Q_{C}^{Other}$ includes components which are assumed to represent fixed fractions of the cell: both $Q_{C}^{Other}$ and its components are independent of light intensity and growth rate (Figure 7, Supplementary Figures 5A, 6).

### Evaluating Allocation to Photosynthetic Apparatus

We first obtain $Q_{C}^{Chl}$ with Equation (17), and then $Q_{C}^{Pro-Pho}$ and $Q_{P}^{Thy}$ from Equations (12) and (13), respectively. $Q_{P}^{Thy}$is stoichiometrically related to $Q_{C}^{Plip-Thy}$:


(27) $$\begin{array}{l} Q_{C}^{Plip-Thy}=Q_{P}^{Thy}Y_{Plip}^{C:P} \end{array}$$

where $Y_{Plip}^{C:P}$ is C:P of phospholipids.

### Evaluating Allocation to Biosynthetic Apparatus

To compute biosynthetic apparatus, we first compute $Q_{C}^{Pro-Bio}$ and $Q_{P}^{RNA}$ from Equations (14) and (15), respectively. $Q_{P}^{RNA}$ is stoichiometrically related to $Q_{C}^{RNA}$:


(28) $$\begin{array}{l} Q_{C}^{RNA}=Q_{P}^{RNA}Y_{RNA}^{C:P} \end{array}$$

where $Y_{RNA}^{C:P}$ is the C:P of RNA.

### Evaluating Maximum Growth Rate, $\mu_{max}^{I}$

$\mu_{max}^{I}$ is the value of growth rate, μ, when all of the flexible component of cellular carbon has been allocated to the growth-related apparatus ($Q_{C}^{Pho}$ and $Q_{C}^{Bio}$) and allocation to C or N storage is negligible. Phosphorus storage is assumed to be polyphosphate, thus not contributing to the carbon budget. Given $Q_{C}^{Sto}$ = 0, and substituting Equations (27, 13, 28, 15, 3, 14, 12, 17) (in this order) into Equation (25) leads to the following quadratic equation in μ:


(29) $$\begin{array}{l} 0=a_{M}\mu^{2}+b_{M}\mu +c_{M} \end{array}$$

where


$$\begin{array}{l} a_{M}=Y_{RNA}^{C:P}A_{RNA}^{P}\left(A_{Pho}A_{Chl}\left(I\right)+A_{Bio}\;\right)\\ b_{M}=\left(1+A_{Pho}+Y_{Plip}^{C:P}A_{Pho}^{P:Chl}\right)A_{Chl}\left(I\right)+A_{Bio}\\ \;+\;Y_{RNA}^{C:P}A_{RNA}^{P}\left(A_{Pho}B_{Chl}\left(I\right)+Q_{C}^{Pro-Other}\right)\\ c_{M}=\left(1+A_{Pho}+Y_{Plip}^{C:P}A_{Pho}^{P:Chl}\right)B_{Chl}\left(I\right)+Q_{C}^{Other}\\ \;+\;Y_{RNA}^{C:P}Q_{P,min}^{RNA}-1 \end{array}$$

Here, the positive solution for μ*-* equals $\mu_{max}^{I}$:


(30) $$\begin{array}{l} \mu_{max}^{I}=\frac{-b_{M}+\sqrt{b_{M}^{2}-4a_{M}c_{M}}}{2a_{M}} \end{array}$$

### Obtaining N:C

N:C is represented by the sum of N from N-containing molecules normalized by cellular C quota:


(31) $$\begin{array}{l} N:C=Q_{N}^{Chl}+Q_{N}^{Pro}+Q_{N}^{RNA}+Q_{N}^{DNA}+Q_{N}^{Nsto} \end{array}$$

Here, we define $Y_{Chl}^{N:C}$, $Y_{Pro}^{N:C}$, $Y_{DNA,}^{N:C}$ and $Y_{RNA}^{N:P}$ as N:C of chlorophyll, protein and DNA and N:P of RNA, respectively (value in Table 1). Using these conversion terms:


(32) $$\begin{array}{l} N:C={Y_{Chl}^{N:C}Q}_{C}^{Chl}+{Y_{Pro}^{N:C}Q}_{C}^{Pro}+Y_{RNA}^{N:P}Q_{C}^{RNA}\\ \;+\;{Y_{DNA}^{N:C}Q}_{C}^{DNA}+Q_{N}^{Nsto} \end{array}$$

Then, by substituting Equations (15, 3, 14, 12, 17) (in this order) into Equation (32), we obtain


(33) $$\begin{array}{l} N:C=a_{N}\mu^{2}+b_{N}\mu +c_{N} \end{array}$$

where


$$\begin{array}{l} a_{N}=Y_{RNA}^{N:P}A_{RNA}^{P}\left(A_{Bio}+A_{Pho}A_{Chl}\left(I\right)\right)\\ b_{N}=(Y_{Chl}^{N:C}A_{Chl}\left(I\right)+Y_{Pro}^{N:C}\left(A_{Bio}+A_{Pho}A_{Chl}(I)\right)\\ \;+\;Y_{RNA}^{N:P}A_{RNA}^{P}\left(A_{Pho}B_{Chl}\left(I\right)+Q_{C}^{Pro-Other}\right))\\ c_{N}=Y_{Chl}^{N:C}B_{Chl}\left(I\right)+Y_{Pro}^{N:C}\left(A_{Pho}B_{Chl}\left(I\right)+Q_{C}^{Pro-Other}\right)\\ \;+\;Y_{RNA}^{N:P}Q_{P,min}^{RNA}+Y_{DNA}^{N:C}Q_{C}^{DNA}+Q_{N}^{Sto} \end{array}$$

When the nitrogen content of RNA is accounted for, we predict a quadratic relationship between N:C and growth rate. However, since the contribution from RNA is small, that from protein dominates and the linear approximation of Equation (19) works well (as seen in the data and un-approximated solution shown in Figure 5A). We define *Q*_*N*_ as N:C and $Q_{N}^{NonSto}$ as N:C without N storage:


(34) $$\begin{array}{l} Q_{N}^{NonSto}=Q_{N}-Q_{N}^{Sto} \end{array}$$

### Obtaining P:C

P:C is represented by the sum of N from N-containing molecules normalized by cellular C quota:


(35) $$\begin{array}{l} P:C=Q_{P}^{RNA}+Q_{P}^{DNA}+Q_{P}^{Thy}+Q_{P}^{Other0}+Q_{P}^{Sto} \end{array}$$

We define $Y_{DNA}^{P:C}$ as P:C of DNA (value in Table 1), which leads to


(36) $$\begin{array}{l} P:C=Q_{P}^{RNA}+Y_{DNA}^{P:C}Q_{C}^{DNA}+Q_{P}^{Thy}+Q_{P}^{Other0}+Q_{P}^{Sto} \end{array}$$

By substituting Equations (15, 3, 14, 13, 12, 17) (in this order) into Equation (36), we obtain


(37) $$\begin{array}{l} P:C=a_{P}\mu^{2}+b_{P}\mu +c_{P} \end{array}$$

where


$$\begin{array}{l} a_{P}=A_{RNA}^{P}\left(A_{Bio}+A_{Pho}A_{Chl}\left(I\right)\;\right)\\ b_{P}=A_{RNA}^{P}\left(A_{Pho}B_{Chl}\left(I\right)+Q_{C}^{Pro-Other}\right)+A_{Pho}^{P:Chl}A_{Chl}\left(I\right)\\ c_{P}=Q_{P,min}^{RNA}+Y_{DNA}^{P:C}Q_{C}^{DNA}+A_{Pho}^{P:Chl}B_{Chl}\left(I\right)+Q_{P}^{Other0}+Q_{P}^{Sto} \end{array}$$

We define *Q*_*P*_ as P:C and $Q_{P}^{NonSto}$ as P:C without P storage:


(38) $$\begin{array}{l} Q_{P}^{NonSto}=Q_{P}-Q_{P}^{Sto} \end{array}$$

Since RNA is the dominant contribution to cellular phosphorus, the relationship between P:C and growth rate is non-linear (Figures 1H, 6A).

### Obtaining N:P

Once we obtain N:C and P:C, N:P can be obtained as follows:


(39) $$\begin{array}{l} N:P=\frac{N:C}{P:C} \end{array}$$

### Evaluating Cellular C Concentration and N and P Storage

There are three types of storage: C in carbohydrates and lipids, N storage assumed to be cyanophycin, and P storage assumed to be polyphosphate. Only C and N storage affect the carbon budget. To compute N and P storage, which we assume accumulate only when each element *is not* limiting, we must first determine which nutrient *is* limiting. To do that, we first compute the carbon-based biomass in the culture under N or P limitation, [*C*_*Cell*_]_*i*_ (mol C m^−3^), where *i* is *N* or *P*, respectively. Carbon biomass, [*C*_*Cell*_]_*i*_, is by definition the product of the cellular carbon quota, *C*_*Cell*_ (mol C cell^−1^), and the cell density, *X*_*i*_ (cell m^−3^):


(40) $$\begin{array}{l} {\left[C_{Cell}\right]}_{i}=C_{Cell}X_{i} \end{array}$$

Under N limitation, the time variation of dissolved inorganic N (or ${\text{NO}}_{3^{-}}$) [*N*] (mol N m^−3^) in the culture is based on the balance between dilution and uptake:


(41) $$\begin{array}{l} \frac{d\left[N\right]}{dt}=D\left({\left[N\right]}_{in}-\left[N\right]\right)-V_{N}C_{Cell}X_{N} \end{array}$$

where *D* is the dilution rate (d^−1^), [*N*]_in_ (mol N m^−3^) is the concentration of dissolved inorganic N (or ${\text{NO}}_{3}^{-}$) in the incoming medium, *V*_*N*_ is the N uptake rate per cellular C (mol N mol C^−1^ d^−1^), *X*_*N*_ (cell m^−3^) is the cell density in the culture under N limitation. We also consider the time variation of *X*_*N*_:


(42) $$\begin{array}{l} \frac{dX_{N}}{dt}=\mu X_{N}-DX_{N} \end{array}$$

At steady state (i.e., d[N]/dt = 0), Equation (41) suggests that


(43) $$\begin{array}{l} {\left[C_{Cell}\right]}_{N}=\frac{D\left({\left[N\right]}_{in}-\left[N\right]\right)}{V_{N}} \end{array}$$

where [*C*_*Cell*_]_*N*_ (mol C m^−3^) (= *C*_*Cell*_*X*_*N*_) is the carbon biomass in the culture under N limitation. The steady state of Equation (42) leads to the following well-known relation for a chemostat at steady state:


(44) $$\begin{array}{l} D=\mu \end{array}$$

To relate *V*_*N*_ to known parameters, we further consider the balance of *Q*_*N*_ :


(45) $$\begin{array}{l} \frac{dQ_{N}}{dt}=V_{N}-\mu Q_{N} \end{array}$$

The steady state of this equation leads to a simple relation between the uptake and consumption of N:


(46) $$\begin{array}{l} V_{N}=\mu Q_{N}^{NonSto} \end{array}$$

as *Q*_*N*_ = $Q_{N}^{NonSto}$ under N limitation (given N storage would be small). By assuming that the amount of limiting nutrient is small relative to that in the incoming medium (here [*N*]_*in*_ >> [*N*]) as in previous chemostat simulations (Inomura et al., 2017, 2018) and as justified by laboratory observations (Laws and Bannister, 1980; Healey, 1985; Bühler et al., 1987), and by substituting Equations (44, 46) into Equation (43), we obtain the simple expression for [*C*_*Cell*_]_*N*_:


(47) $$\begin{array}{l} {\left[C_{Cell}\right]}_{N}=\frac{{\left[N\right]}_{in}}{Q_{N}^{NonSto}} \end{array}$$

We follow the same procedures above (Equations 41–47) by replacing N with P to obtain an expression for the carbon biomass in the culture under P limitation [_*C*_*Cell*_]*P*_ (mol C m^−3^):


(48) $$\begin{array}{l} {\left[C_{Cell}\right]}_{P}=\frac{{\left[P\right]}_{in}}{Q_{P}^{NonSto}} \end{array}$$

Here [*P*]_*in*_ (mol m^−3^) is the concentration of dissolved inorganic P (${\text{PO}}_{4}^{3-}$) in the incoming medium.

We assume that the limiting resource is that which gives the smallest cellular C concentration in the culture. For example, when [*C*_*Cell*_]_*N*_ < [*C*_*Cell*_]_*P*_, the culture is limited by N since this relationship with Equations (47) and (48) leads to the following equation:


(49) $$\begin{array}{l} \frac{{\left[N\right]}_{in}}{{\left[P\right]}_{in}}<\frac{Q_{N}^{NonSto}}{Q_{P}^{NonSto}} \end{array}$$

showing that the input N:P (left hand side) is lower than required N:P (right hand side). On the other hand, when [*C*_*Cell*_]_*N*_ > [*C*_*Cell*_]_*P*_, input N:P is higher than required N:P indicating excess N and thus P limitation. Once the nutrient limitation is determined, we define the actual cellular C concentration in the culture [*C*_*Cell*_] (cell^−1^ m^−3^):


(50) $$\begin{array}{l} \left[C_{Cell}\right]=\min\left({\left[C_{Cell}\right]}_{N},\;{\left[C_{Cell}\right]}_{P}\right) \end{array}$$

With this equation, we have simulated the relationship between biomass, [*C*_*Cell*_], and growth rate, μ, which reveals a decreasing trend with dilution rates, capturing the observation (Healey, 1985; Supplementary Figure 7).

If the culture is N limited, there is an excess of P, which could be stored in the cell. To determine the potential level of cellular P based on the P availability ($Q_{P}^{Pot}$) (mol P mol C^−1^), we follow the same procedure as when determining [*C*_*Cell*_]_*N*_ from Equations (41)–(47), by using P instead of N, except for using *X*_*N*_ and [*C*_*Cell*_]_*N*_ obtained previously:


(51) $$\begin{array}{l} Q_{P}^{Pot}=\frac{{\left[P\right]}_{in}}{{\left[C_{Cell}\right]}_{N}} \end{array}$$

Then we compare this potential quota with the maximum capacity of cellular P ($Q_{P}^{max}$) (mol P mol C^−1^) (mol P mol C^−1^) and define *Q*_*P*_:


(52) $$\begin{array}{l} Q_{P}=\min\left(Q_{P}^{Pot},Q_{P}^{max}\right) \end{array}$$

and by rearranging Equation (38) we determine $Q_{P}^{Sto}$:


(53) $$\begin{array}{l} Q_{P}^{Sto}=Q_{P}-Q_{P}^{NonSto} \end{array}$$

Under P limitation, N storage is accumulated since excess N is available. By following the same steps as above with reversed N and P and by rearranging Equation (34), we obtain the following relations:


(54) $$\begin{array}{l} Q_{N}^{Pot}=\frac{{\left[N\right]}_{in}}{{\left[C_{Cell}\right]}_{P}} \end{array}$$

(55) $$\begin{array}{l} Q_{N}=\min\left(Q_{N}^{Pot},Q_{N}^{max}\right) \end{array}$$

(56) $$\begin{array}{l} Q_{N}^{Sto}=Q_{N}-Q_{N}^{NonSto} \end{array}$$

where $Q_{N}^{Pot}$ (mol N mol C^−1^) is the potential cellular quota of N normalized by C based on the N availability and the cellular C determined by P limitation and $Q_{N}^{max}$ (mol N mol C^−1^) is the maximum cellular capacity of N normalized by cellular C. We note that in most cases, $Q_{N}^{Pot}$ > $Q_{N}^{max}$ (or $Q_{P}^{Pot}$ > $Q_{P}^{max}$). However, when the N:P ratio without storage molecules (i.e., $Q_{N}^{NonSto}$/$Q_{P}^{NonSto}$) is close to the N:P ratio of the resource (i.e., [*N*]_*in*_/[*P*]_*in*_), the excess nutrient is relatively small and $Q_{N}^{Pot}$ < $Q_{N}^{max}$ (or $Q_{P}^{Pot}$ < $Q_{P}^{max}$) can occur (e.g., at the light intensity of 12 μmol m^−2^ s^−1^ in Supplementary Figure 4A).

The data and model together reveal that N and P “storage” work differently (Supplementary Figure 4) in *S. linearis* (Healey, 1985). Under nitrogen limitation, the total phosphorus quota per carbon appears relatively constant while under P-limitation, N storage appears to be relatively constant. Hence, to model the storage contributions, we have imposed maximum total P quota per carbon ($Q_{P}^{max}$) (mol P mol C^−1^) and maximum N storage per carbon ($Q_{N}^{Sto,max}$) (mol N mol C^−1^); thus $Q_{N}^{max}=Q_{N}^{Sto,max}+Q_{N}^{NonSto}$. In other words, maximum P storage depends on the level of other P molecules while maximum N storage is independent from the level other N molecules. These simple assumptions allow us reproduce N:C under P limitation and P:C under N limitation (Supplementary Figure 4) as well as N:P (Figure 9).

This model of the storage pools is simple and logical, yet still somewhat *ad hoc* and empirically driven. Because of the importance of storage of the non-limiting element, prediction of the N:P ratio depends on this. Clearer understanding of the dynamics of the storage pools will be necessary to provide more mechanistic models. Laboratory data which resolves the macromolecular pools in sufficient detail would aid this effort. Interpretations of the Redfieldian N:P ratio as a homeostatic protein:RNA ratio (Loladze and Elser, 2011), while revealing the central controls, do not necessarily reflect these important “storage” dynamics (Supplementary Figure 4).

N storage has an associated C (e.g., cyanophycin), which can be obtained from a given elemental ratio of N storage:


(57) $$\begin{array}{l} Q_{C}^{Nsto}=Y_{Nsto}^{C:N}Q_{N}^{Sto} \end{array}$$

### Evaluating C Storage

The difference between the total cellular C and computed sum of macromolecular C is assumed to be C storage:


(58) $$\begin{array}{l} Q_{C}^{Csto}=1-Q_{C}^{Chl}-Q_{C}^{Pro}-Q_{C}^{RNA}-Q_{C}^{DNA}-Q_{C}^{Plip-Thy}\\ \;-\;Q_{C}^{Nsto}-Q_{C}^{Other0} \end{array}$$

### Parameter Estimation

Elemental stoichiometry of each molecule and some parameters are assumed based on available information (Supplementary Table 4). As a result, there are 11 parameters that need to be estimated from the data: *m*, $v_{I}^{max}$, *A*_*I*_, *A*_*Pho*_, *A*_*Bio*_, $Q_{C}^{Pro-Other}$, $A_{RNA}^{P}$, $Y_{Pho}^{P:Chl}$, $Q_{P}^{Other0}$, $Q_{N}^{Sto,max}$, $Q_{C}^{Other0}$ (values in Supplementary Table 5). To estimate parameter values that best fit experimental data, we adapt the Metropolis-Hastings algorithm (Metropolis et al., 1953; Hastings, 1970; Omta et al., 2017), a Markov Chain Monte Carlo method. The implementation of the algorithm is described in detail below. We begin by using the Metropolis-Hastings algorithm to fit Equation (17) to the chlorophyll:C vs. μ data for *S. linearis* (Healey, 1985; Figure 4), estimating the values for 3 chlorophyll related parameters (*m*, $v_{I}^{max}$, *A*_*I*_). We estimate these independently of the other parameters, which do not influence chlorophyll. Next, we estimate nitrogen related parameters (*A*_*Pho*_, *A*_*Bio*_, $Q_{C}^{Pro-Other}$, $Q_{N}^{Sto,max}$) and the parameter $Q_{C}^{Other0}$, by fitting Equations (33, 50, 30) to the observed data for N:C vs. μ under both N and P limitations (Figure 5A, Supplementary Figure 4Ai) for C concentration vs. μ under N limitation (Supplementary Figure 7A) and for $\mu_{max}^{I}$ vs. *I* under both N and P limitations (Figure 8A, Supplementary Figure 5B), respectively. Finally, we estimate the remaining three parameters ($A_{RNA}^{P}$, $Y_{Pho}^{P:Chl}$, $Q_{P}^{Other0}$) by fitting Equations (50, 37) to data for the concentration of cellular C (Supplementary Figure 7B) and for P:C (Figure 6A) under P limitation.

For *Pavlova lutheri* (Chalup and Laws, 1990) and *Skeletonema costatum* (Sakshaug and Andersen, 1989), we use the data of chlorophyll (Supplementary Figure 2i) to estimate *m*, $v_{I}^{max}$, *A*_*I*_, and N:C (Supplementary Figure 2ii) and $\mu_{max}^{I}$ (Figures 8B,C) to estimate *A*_*Pho*_, *A*_*Bio*_, $Q_{C}^{Pro-Other}$, $Q_{C}^{Other0}$. For these cases, certain parameters are adopted from *S. linearis* since the experimental information is less comprehensive and appropriate data is not available to constrain them; these other parameters have limited influence on the specific model results illustrated here (i.e., Chl:C, N:C, $\mu_{max}^{I}$).

#### Algorithm

The model is first solved with an initial set of parameters, which are determined by manually tuning values until model solutions are reasonably consistent with the data. The algorithm then proceeds in a series of steps (a “chain” of steps) that introduce random perturbations to the parameter values. It finds a set of values that provide a good fit to the data by keeping new parameter values that fit the data well (the new parameters become the “current” state of the parameters), and usually discarding others. That is, if the new parameters fit the data much more poorly than the “current” state, there is a high probability they will be rejected. In aggregate, the algorithm evaluates many combinations of parameters in the search for globally optimal solutions.

#### Evaluating the Fit Between Model and Data

For a given set of parameters (*Pset*), beginning with the initial set, we compute a measure of the fit between the model and data. We use the sum of squared errors for each data set between the model (with parameters *Pset*) and data points, given by


(59) $$\begin{array}{l} Error_{j}^{k}=\underset{i}{\sum }\frac{{\left(Data\left(\mu_{i}^{k},I_{i}^{k}\right)-Model\left(\mu_{i}^{k},I_{i}^{k},Pset_{j}\right)\right)}^{2}}{2\sigma_{k}^{2}} \end{array}$$

where *i* and *k* indicate the *i*th measurement of *k*th data set, *j* is the *j*th iteration (initial step, *j* = 1), $Data\left(\mu_{i}^{k},I_{i}^{k}\right)$ are data for different growth rates μ and light intensities *I*, $Model\left(\mu_{i}^{k},I_{i}^{k},Pset_{j}\right)$is the model estimated for the same μ and *I* with a parameter set *Pset*_*j*_, and σ_*k*_ is an estimate of the measurement error of the *k*th dataset. We estimate these values based on the magnitude of scatter among measurements that are made under similar experimental conditions. Once we obtain an error value for each dataset, we normalize the data with the number of data *n*_*k*_ and add them up to obtain the error covering all the datasets:


(60) $$\begin{array}{l} Error_{j}=\underset{k}{\sum }\frac{Error_{j}^{k}}{n_{k}} \end{array}$$

This normalization by *n*_*k*_ is intended to give similar weight to data sets with different numbers of observations and resulted in slightly improved model-data fit for $\mu_{max}^{I}$ at high light intensities while keeping other model outputs visually unchanged.

#### Iteration

At each step in the chain, we generate a new parameter set with small random perturbations of the previous set (in this study, within the range of ±20%). In *Pset*_*j*_ we only accept positive values and chlorophyll related parameters less than certain values (mostly ~5 times of the estimated values), since values outside of these ranges are less likely. We then compare the fit of the *j*th proposed parameter set to the current set, based on the likelihood ratio, given by


(61) $$\begin{array}{l} Ratio_{j}=\exp\left(-Error_{j}+Error_{Current}\right) \end{array}$$

Once we obtain the likelihood ratio, we generate a uniform random number (*Random*) between 0 and 1 and compare it with *Ratio*_*j*_. If *Ratio*_*j*_ > *Random*, we update the current parameter set to be the *j*th set. Therefore, if *Error*_*j*_ is smaller than *Error*_*Current*_ (i.e., the model with *Pset*_*j*_ fits better than the current model), the current parameters are updated to *Pset*_*j*_. However, if *Error*_*Current*_ is smaller than *Error*_*j*_, the *j*th state will be accepted with a probability that declines as a function of the difference between the error terms. This means good parameter sets tend to be kept, but the acceptance of poorer sets provides a mechanism to get out of the local maxima. After many steps (10^6^ steps), we identified the parameter set that gives the smallest errors between model and data.

## Data Availability Statement

The model code for this study can be found in GitHub https://github.com/ag105020/Phyto1 (10.5281/zenodo.1203718).

## Author Contributions

KI, JB, and MF designed the study. KI, AO, DT, and MF gathered data. KI developed a model and led the project. KI parameterized the model with help of AO, DT, and JB. KI, CD, and MF acquired funding. CD supervised KI. MF advised KI, AO, and DT. All the authors wrote the manuscript.

### Conflict of Interest

The authors declare that the research was conducted in the absence of any commercial or financial relationships that could be construed as a potential conflict of interest.
