# Corrigendum: A mechanistic model of macromolecular allocation, elemental stoichiometry, and growth rate in phytoplankton

**DOI:** 10.3389/fmicb.2024.1486795

**Published:** 2024-10-18

**Authors:** Keisuke Inomura, Anne Willem Omta, David Talmy, Jason Bragg, Curtis Deutsch, Michael J. Follows

**Affiliations:** ^1^School of Oceanography, University of Washington, Seattle, WA, United States; ^2^Department of Earth, Atmospheric and Planetary Sciences, Massachusetts Institute of Technology, Cambridge, MA, United States; ^3^Department of Microbiology, University of Tennessee, Knoxville, Knoxville, TN, United States; ^4^National Herbarium of New South Wales, The Royal Botanic Gardens and Domain Trust, Sydney, NSW, Australia

**Keywords:** phytoplankton, elemental stoichiometry, growth rate, macromolecule, photosynthesis, protein, RNA, nutrient storage

In the published article, there were mistakes in the values in Table 1, particularly in the elemental ratio values for protein, RNA and DNA as published. Here, we provide a revised table with the corrected values (Table 1). After the value correction, we reoptimized the parameters (Supplementary Table 5), which produce nearly identical results with the original version (examples in Figure C1).

**Figure C1:**
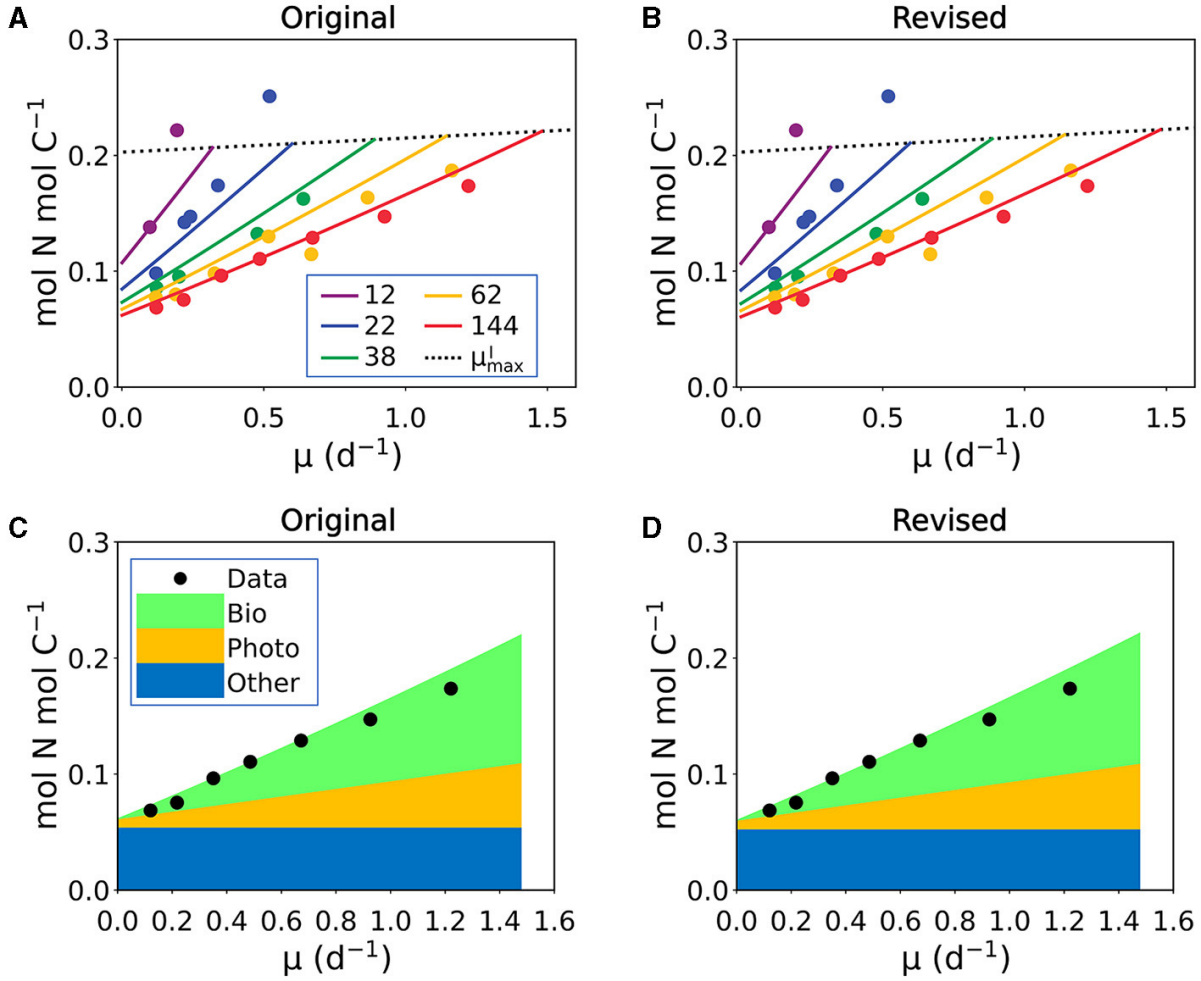
Example comparison between the versions with original parameters and revised parameters. The results and data are N:C under N limitation. Plots are model-data comparisons, where lines and stack plots are model outputs and points are data (Healey et al., 1985). The model outputs in **(A, C)** are based on the original parameters and **(B, D)** are based on the revised parameters. As shown, the original and revised versions produce nearly identical output. See Figure 5 capion in the original publication for details.

**Table 1:** Elemental stoichiometry of some macromolecules.

**Molecule**	**C:N:P**	**Explanation**
Chlorophyll	55:4:0	Chlorophyll A
Protein	3.82:1:0	Average value based on (Brown, 1991)
RNA	9.5:3.78:1	Based on CG = 0.563: *Synechococcus spp*.^*^
DNA	9.72:3.78:1	Based on CG = 0.563: *Synechococcus spp*.^*^
P lipid	40:0:1	Phosphatidylglycerol with C16 fatty acids
C store	1:0:0	Carbohydrate and non-phospholipid
N store	2:1:0	Cyanophycin
P store	0:0:1	Polyphosphate

* GC% [http://www.ncbi.nlm.nih.gov/genome/13522 (accessed December 13, 2018)].

The authors apologize for this error and state that this does not change the scientific conclusions of the article in any way. The original article has been updated with revised Table 1 and Supplementary Table 5.

