# Supplementary material for: A Mechanistic Model of Macromolecular Allocation, Elemental Stoichiometry, and Growth Rate in Phytoplankton
Source: Front Microbiol. 2020 Feb 28;11:86. doi: 10.3389/fmicb.2020.00086 (PMC7093025; doi:10.3389/fmicb.2020.00086)
Supplement: Supplementary file 1 [file Data_Sheet_1.pdf]

Supplementary Figures:

A mechanistic model of macromolecular allocation, elemental stoichiometry and growth rate in phytoplankton

Keisuke Inomura, Anne Willem Omta, David Talmy, Jason Bragg, Curtis Deutsch and Michael J. Follows

Contents:

Supplementary Figures 1–7

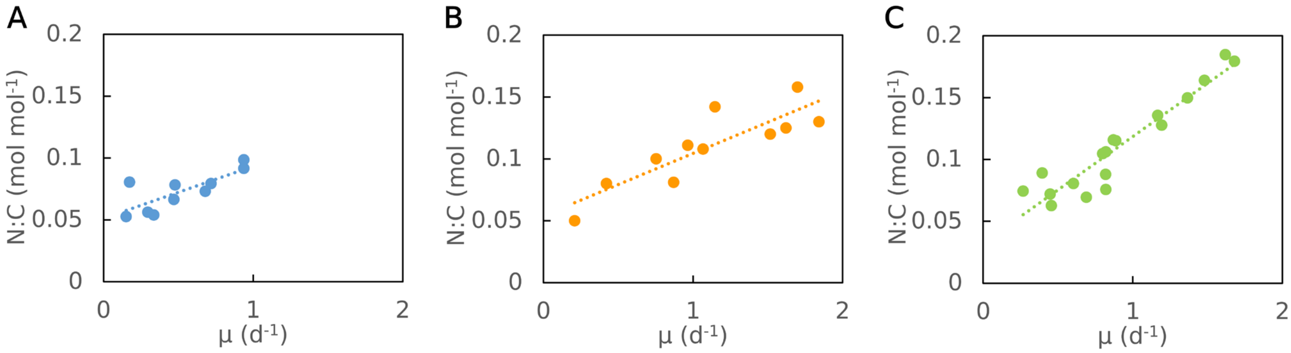

Supplementary Figure 1. Laboratory data of growth rate dependence of N:C in three phytoplankton species. **(A)** *Thalassiosira fluviatilis* (Laws and Bannister, 1980). **(B)** *Cyclotella nana* (Caperon and Meyer, 1972). **(C)** *Selenastrum minutum* (Elrifi and Turpin, 1985). Points are data and curves represent regression lines.

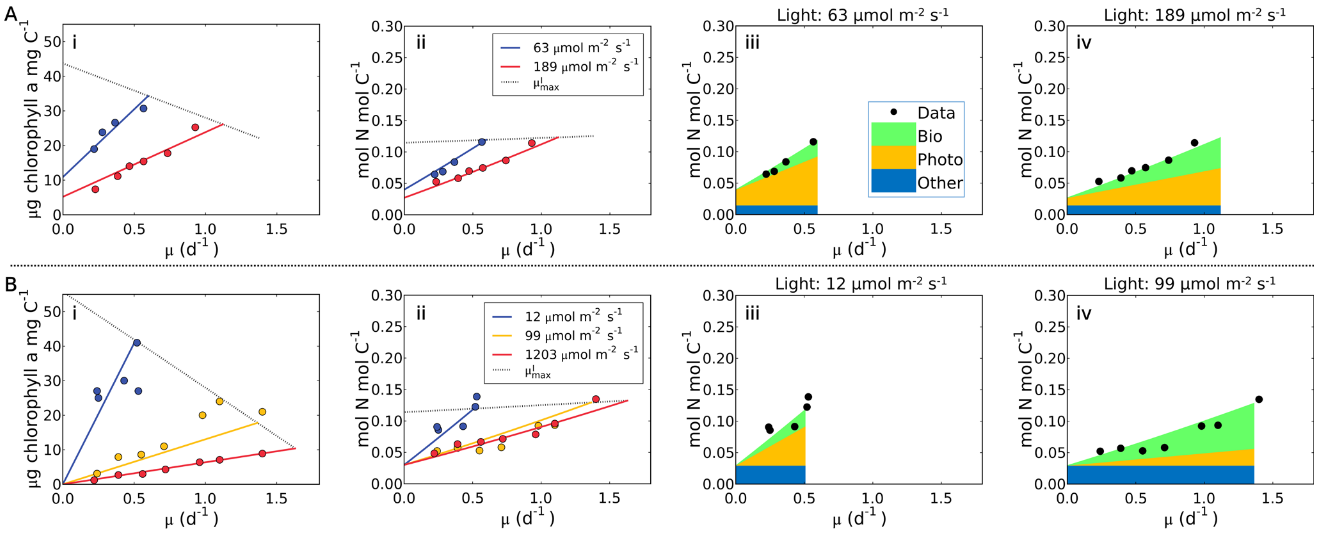

Supplementary Figure 2. Chlorophyll and N:C and macromolecular contribution to N:C for two marine algae. **(A)** *Pavlova lutheri* (Chalup and Laws, 1990). **(B)** *Skeletonema costatum* (Sakshaug and Andersen, 1989). **(i)** Chlorophyll per carbon for various irradiances (see **(ii)** for light values). **(ii)** N:C for various light intensities. For **(i)** and **(ii)**, points are data and curves are model outputs. Dotted lines indicate μ^I_{max}. In **(B)** model/data of 71 µmol m^{-2} s^{-1} (light) are not included, as the data shows unusually high values potentially due to the use of different lamps (Sakshaug and Andersen, 1989). **(iii)** and **(iv)** Macromolecular contribution in N, normalized by cellular C for different light intensities; points are data and color contours are models (see **(Aiii)** for color definitions: *Bio*, Biosynthetic protein + RNA (dominated by Biosynthetic protein); *Photo*, chlorophyll + photosynthetic protein; *Other*, other molecules.).

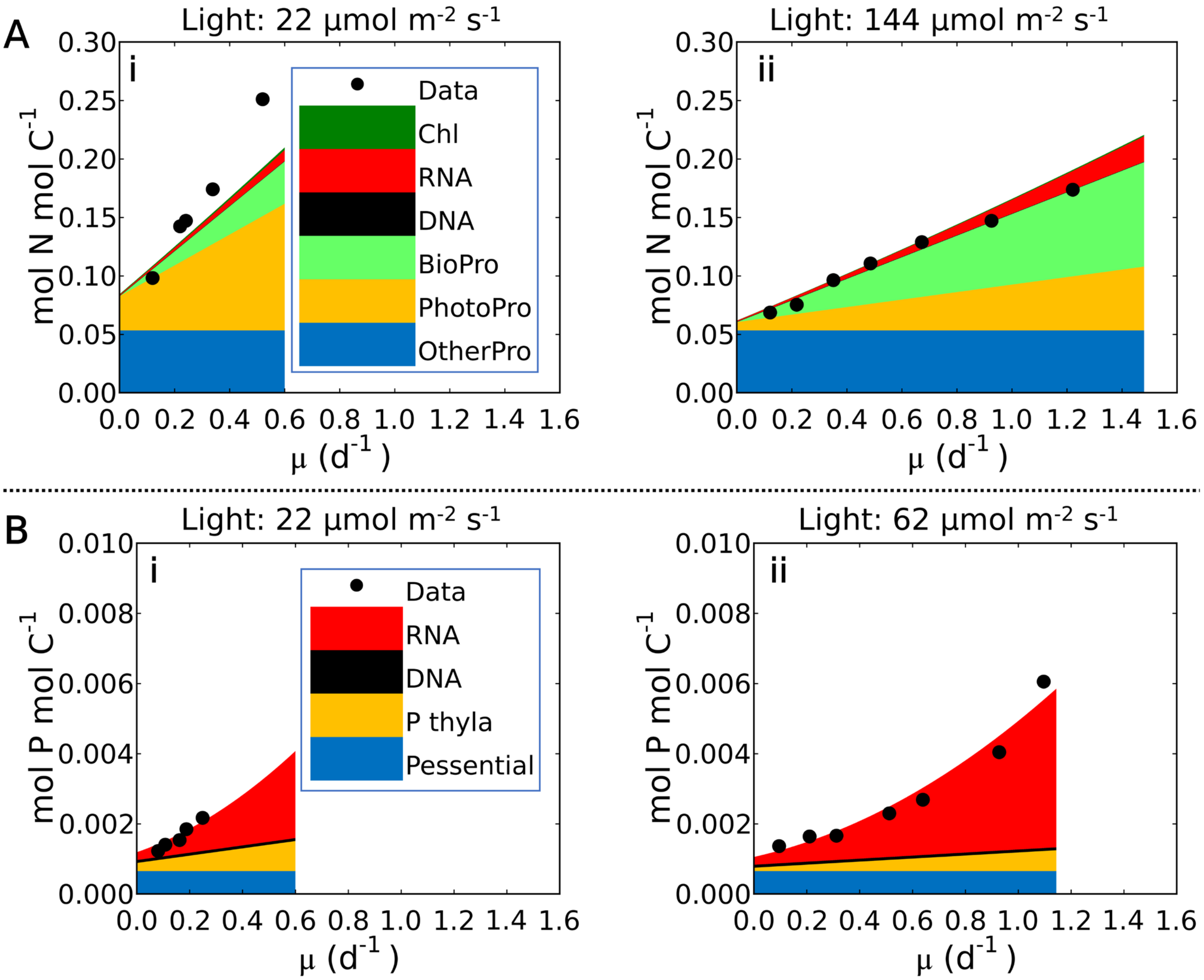

Supplementary Figure 3. Detailed macromolecular contribution to N **(A)** and P **(B)** normalized by C for various growth rates and light intensities. The total value is compared to data. *Chl*, chlorophyll; *BioPro*, biosynthetic proteins; *PhotoPro*, photosynthetic proteins; *OtherPro*, other proteins; *P thyla*, P from thylakoid membrane; *Pessential*, essential P quota.

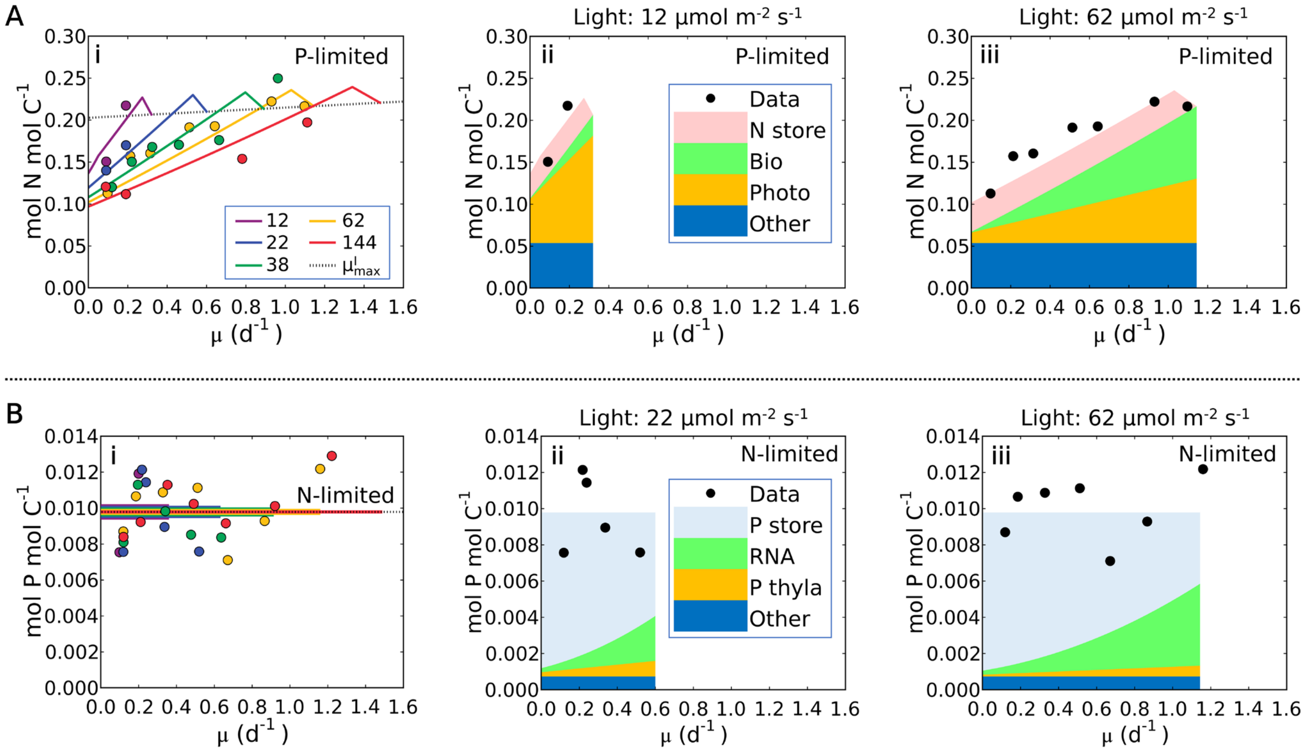

Supplementary Figure 4. Model data comparison of N:C and P:C and model prediction of macromolecular allocation of *Synechococcus linearis* when excess nutrients are available. **(A)** N:C under P limitation. **(B)** P:C under N limitation. **(i)** N:C **(A)** and P:C **(B)** for different light intensities; light intensities (μmol m^{-2} s^{-1}) are in the legend in (Ai). Curves are model results and points are data (Healey, 1985). Dotted lines represent μ^{I}_{max} at various light intensities; high μ^{I}_{max} for higher light intensity. **(ii)**, **(iii)** Macromolecular allocation for N **(A)** and P **(B)** normalized by cellular C for different light intensities; 12 (μmol m^{-2} s^{-1}) for **(Aii)** 22 (μmol m^{-2} s^{-1}) for **(Bii)** and 62 (μmol m^{-2} s^{-1}) for **(iii)**. Black points are data for total values (Healey, 1985). See (**Aii**) and (**Bii**) for color definitions of macromolecular allocations: *N store*, N storage; *P store*, P storage; *Bio*, Biosynthetic protein + RNA; *Photo*, chlorophyll + photosynthetic protein; P thyla, thylakoid P; *Other*, other molecules. *Bio* in N allocation is mostly biosynthetic protein and *Photo* in N allocation is dominated by photosynthetic protein.

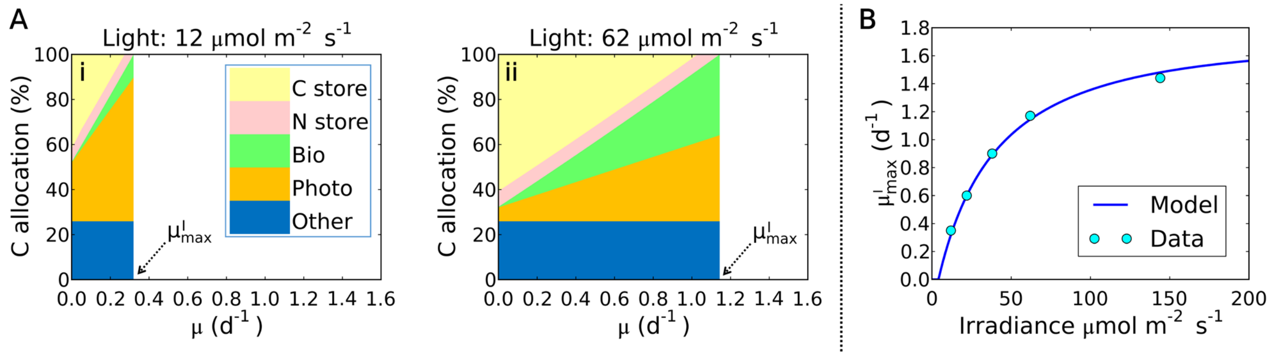

Supplementary Figure 5. Carbon allocation and nutrient replete growth rate (μ^{I}_{max}) as a function of irradiance under P limitation. **(A)** Simulated carbon allocation of *Synechococcus linearis* for various μ and at light intensities of 12 **(i)** and 62 µmol m^{-2} s^{-1} **(ii)**. See **(i)** for color definitions: *C store*, C storage; *Bio*, Biosynthetic protein + RNA; *Photo*, Chlorophyll + Photosynthetic protein + P-lipid in thylakoid membranes; *Other*, other constant molecules. *Bio* and *Photo* are dominated by biosynthetic and photosynthetic proteins, respectively. Where C store becomes zero indicates μ^{I}_{max}. **(B)** μ^{I}_{max} to light relationships for *Synechococcus linearis*. Modeled μ^{I}_{max} is compared to data (Healey, 1985).

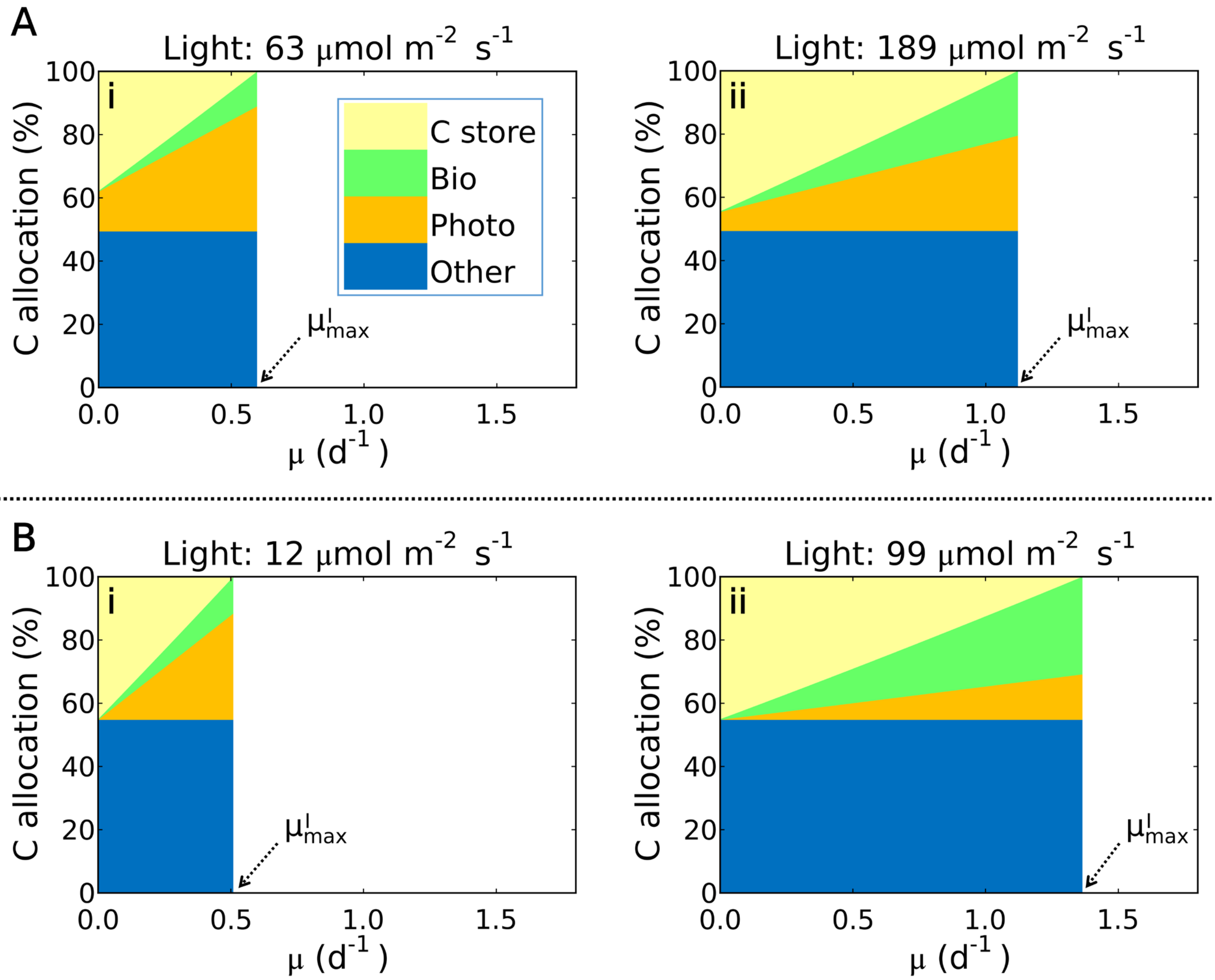

Supplementary Figure 6. Simulated carbon allocation of different light intensities for two marine phytoplankton. **(A)** *Pavlova lutheri* (Chalup and Laws, 1990) at light intensities of 63 **(i)** and 189 μmol m^{-2} s^{-1} **(ii)**. **(B)** *Skeletonema costatum* (Sakshaug and Andersen, 1989) at light intensities of 12 **(i)** and 99 μmol m^{-2} s^{-1} **(ii)**. See (Ai) for color definitions: *Bio*, Biosynthetic protein + RNA; *Photo*, Chlorophyll + Photosynthetic protein + P-lipid in thylakoid membranes; *Other*, other constant molecules. *Bio* and *Photo* are dominated by biosynthetic and photosynthetic proteins, respectively. Where C store becomes zero indicates μ^I_{max}.

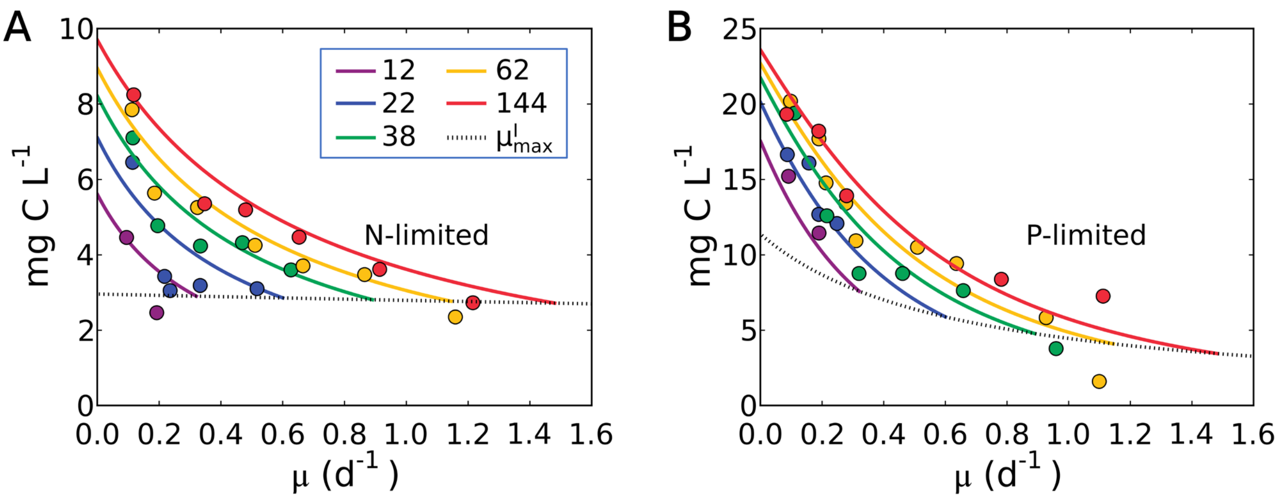

Supplementary Figure 7. Model data comparison of biomass C concentration in a culture. **(A)** Under N limitation. **(B)** Under P limitation. Curves: model. Points: data of *Synechococcus linearis* (Healey, 1985). Dotted black line indicates values at μ^I_{max} for various light intensities. Numbers in the legend show light intensities in (μmol m^{-2} s^{-1}).

References

Caperon, J., and Meyer, J. (1972). Nitrogen-limited growth of marine phytoplankton-I. Changes in population characteristics with steady-state growth rate. *Deep Sea Res.* 19, 601–618.

Chalup, M. S., and Laws, E. A. (1990). A test of the assumptions and predictions of recent microalgal growth models with the marine phytoplankter *Pavlova lutheri*. *Limnol. Oceanogr.* 35, 583–596. doi:10.4319/lo.1990.35.3.0583.

Elrifi, I. R., and Turpin, D. H. (1985). Steady-state luxury consumption and the concept of optimum nutrient ratios: A study with phosphate nitrate limited *Selenastrum minutum* (Chlorophyta). *J. Phycol.* 21, 592–602.

Healey, F. P. (1985). Interacting effects of light and nutrient limitation on the growth rate of *Synechococcus linearis* (Cyanophyceae). *J. Phycol.* 21, 134–146.

Laws, E. A., and Bannister, T. T. (1980). Nutrient- and light-limited growth of *Thalassiosira fluviatilis* in continuous culture, with implications for phytoplankton growth in the ocean. *Limnol. Oceanogr.* 25, 457–473. doi:10.4319/lo.1980.25.3.0457.

Sakshaug, E., and Andersen, K. (1989). A steady state description of growth and light absorption in the marine planktonic diatom *Skeletonema costatum*. *Limnol. Oceanogr.* 34, 198–205.
