# Supplementary material for: A Mechanistic Model of Macromolecular Allocation, Elemental Stoichiometry, and Growth Rate in Phytoplankton
Source: Front Microbiol. 2020 Feb 28;11:86. doi: 10.3389/fmicb.2020.00086 (PMC7093025; doi:10.3389/fmicb.2020.00086)
Supplement: Supplementary file 2 [file Data_Sheet_2.pdf]

Supplementary Tables:

A mechanistic model of macromolecular allocation, elemental stoichiometry and growth rate in phytoplankton

Keisuke Inomura, Anne Willem Omta, David Talmy, Jason Bragg, Curtis Deutsch and Michael J. Follows

Contents:

Supplementary Tables 1–5

Supplementary Table 1. R^2 values for Chl:C for the data in Figure 1

Panel	Light intensity (μmol m^{-2} s^{-1})	R^2
A	63	0.903
	189	0.973
B	12	0.355
	71	0.521
	99	0.874
	1203	0.989
C	12	0.521
	22	0.856
	38	0.879
	62	0.954
	144	0.906

Mean and standard deviation of R^2 are 0.794 and 0.209.

Supplementary Table 2. R^2 values for N:C for the data in Figure 1 and Supplementary Figure 1

Figure 1		
Panel	Light intensity (μmol m^{-2} s^{-1}) or assigned alphabet	R^2
D	63	0.993
	189	0.957
E	12	0.723
	71	0.779
	99	0.877
	1203	0.892
F	22	0.993
	38	0.993
	62	0.942
	144	0.989
G	a	0.310
	b	0.387
	c	0.985
	d	0.761
	e	0.945
	f	0.936

Supplementary Figure 1	
Panel	R^2
A	0.607
B	0.746
C	0.889

Data with only two data points are not considered. Mean and standard deviation of R^2 are 0.827 and 0.197.

Supplementary Table 3. Compilation of the final equations for the full model.

$$Q_C^{Chl} = A_{Chl}(I)\mu + B_{Chl}(I) \quad \text{[eq. 17]}$$

where $A_{Chl}(I) = (1+E)/v_I(I)$ and $B_{Chl}(I) = m/v_I(I)$

$$\text{where } v_I(I) = v_I^{max}(1 - e^{-A_I I}) \quad \text{[eq. 11]}$$

$$\mu_{max}^{I} = \frac{-b_M + \sqrt{b_M^2 - 4a_M c_M}}{2a_M} \quad \text{[eq. 30]}$$

where

$$a_M = Y_{RNA}^{C:P} A_{RNA}^{P} \left(A_{Pho} A_{Chl}(I) + A_{Bio}\right)$$

$$b_M = \left(1 + A_{Pho} + Y_{Plip}^{C:P} A_{Pho}^{P:Chl}\right) A_{Chl}(I) + A_{Bio} + Y_{RNA}^{C:P} A_{RNA}^{P} \left(A_{Pho} B_{Chl}(I) + Q_C^{Pro-Other}\right)$$

$$c_M = \left(1 + A_{Pho} + Y_{Plip}^{C:P} A_{Pho}^{P:Chl}\right) B_{Chl}(I) + Q_C^{Other} + Y_{RNA}^{C:P} Q_{P,min}^{RNA} - 1$$

$$N{:}C = a_N \mu^2 + b_N \mu + c_N \quad \text{[eq. 33]}$$

where

$$a_N = Y_{RNA}^{N:P} A_{RNA}^{P} \left(A_{Bio} + A_{Pho} A_{Chl}(I)\right)$$

$$b_N = \left(Y_{Chl}^{N:C} A_{Chl}(I) + Y_{Pro}^{N:C}\left(A_{Bio} + A_{Pho} A_{Chl}(I)\right) + Y_{RNA}^{N:P} A_{RNA}^{P}\left(A_{Pho} B_{Chl}(I) + Q_C^{Pro-Other}\right)\right)$$

$$c_N = Y_{Chl}^{N:C} B_{Chl}(I) + Y_{Pro}^{N:C}\left(A_{Pho} B_{Chl}(I) + Q_C^{Pro-Other}\right) + Y_{RNA}^{N:P} Q_{P,min}^{RNA} + Y_{DNA}^{N:C} Q_C^{DNA} + Q_N^{Sto}$$

$$P{:}C = a_P \mu^2 + b_P \mu + c_P \quad \text{[eq. 37]}$$

$$N{:}P = \frac{N{:}C}{P{:}C} \quad \text{[eq. 39]}$$

where

$$a_P = A_{RNA}^{P}\left(A_{Bio} + A_{Pho} A_{Chl}(I)\right)$$

$$b_P = A_{RNA}^{P}\left(A_{Pho} B_{Chl}(I) + Q_C^{Pro-Other}\right) + A_{Pho}^{P:Chl} A_{Chl}(I)$$

$$c_P = Q_{P,min}^{RNA} + Y_{DNA}^{P:C} Q_C^{DNA} + A_{Pho}^{P:Chl} B_{Chl}(I) + Q_P^{Other0} + Q_P^{Sto}$$

$$[C_{Cell}] = \min([C_{Cell}]_N, [C_{Cell}]_P) \quad \text{[eq. 50]}$$

$$\text{where } [C_{Cell}]_N = [N]_{in}/Q_N^{NonSto} \quad \text{[eq. 47]}$$

$$\text{and } [C_{Cell}]_P = [P]_{in}/Q_P^{NonSto} \quad \text{[eq. 48]}$$

$$\text{where } Q_N^{NonSto} = Q_N - Q_N^{Sto} \quad \text{[eq. 34]}$$

$$\text{and } Q_P^{NonSto} = Q_P - Q_P^{Sto} \quad \text{[eq. 38]}$$

See "Evaluating cellular C concentration and N and P storage" in Methods section for the estimation of Q_N^{Sto} and Q_P^{Sto}.

Supplementary Table 4. Values used for fixed constants

Parameter	Value	Unit
E	7.74×10^{-1}	Dimensionless
Q_C^{DNA}	9.41×10^{-4}	mol C mol C^{-1}
$Q_{P,min}^{RNA}$	2.23×10^{-4}	mol P mol C^{-1}
Q_P^{max}	9.79×10^{-3}	mol P mol C^{-1}
$[P]_{IN}$	2.00×10^{-3} for P limiting and 2.00×10^{-2} for N limiting culture	mol P m^{-3}
$[N]_{IN}$	2.00×10^{-1} for P limiting and 5.00×10^{-2} for N limiting culture	mol N m^{-3}

E is estimated with mass, electron and energy balances (Rittmann and McCarty, 2001) with suggested energy transfer efficiency of 0.6 and stoichiometry $C_5H_7O_2N_1P_{1/30}$ with NO_3^- and PO_4^{3-} for N and P sources respectively. Q_C^{DNA} is estimated based on gene size of *Synechococcus* sp. (http://www.ncbi.nlm.nih.gov/genome/13522; accessed 12/13/2018) and cellular carbon quota of *Synechococcus linearis* under N-limitation (Healey, 1985). $Q_{P,min}^{RNA}$ is estimated based on RNA:DNA ratio of *E. coli* at zero growth (Bremer and Dennis, 1996). Q_P^{max} is based on the averaged value from N limited culture in Healey's paper (Healey, 1985). $[P]_{IN}$ and $[N]_{IN}$ represent Healey's experiment (Healey, 1985).

Supplementary Table 5. Values used for adjustable parameters

Parameter	*Synechococcus linearis* (Healey, 1985)	*Pavlova lutheri* (Chalup and Laws, 1990)	*Skeletonema costatum* (Sakshaug and Andersen, 1989)	Unit
m	3.93×10^{-1}	4.96×10^{-1}	5.18×10^{-6}	d^{-1}
P_{Chl}^{max}	2.77×10^{2}	1.81×10^{2}	3.77×10^{2}	mol C (mol C Chl)$^{-1}$ d^{-1}
A_I	8.63×10^{-3}	6.57×10^{-3}	6.75×10^{-3}	μmol^{-1} m^{2} s
A_{Photo}	1.38×10^{1}	1.13×10^{1}	7.45×10^{0}	mol C (mol C Chl)$^{-1}$
A_{Bio}	2.36×10^{-1}	1.37×10^{-1}	1.69×10^{-1}	mol C mol C^{-1} d
$C_{Protein}^{Other}$	1.99×10^{-1}	5.46×10^{-2}	1.15×10^{-1}	mol C mol C^{-1}
A_{RNA}^{P}	4.95×10^{-3}	4.95×10^{-3}	4.95×10^{-3}	mol P mol C^{-1} d
$Y_{Photo}^{P:Chl}$	2.81×10^{-2}	2.81×10^{-2}	2.81×10^{-2}	mol P (mol C Chl)$^{-1}$
$P_{Essential}$	6.57×10^{-4}	6.57×10^{-4}	6.57×10^{-4}	mol P mol C^{-1}
N_{Store}^{max}	3.41×10^{-2}	3.41×10^{-2}	3.41×10^{-2}	mol N mol C^{-1}
$C_{Essential}$	1.52×10^{-1}	5.08×10^{-1}	4.98×10^{-1}	mol C mol C^{-1}

References

Bremer, H., and Dennis, P. (1996). Modulation of chemical composition and other parameters of the cell by growth rate. In: Neidhardt F (eds). *Escherichia coli* and *Salmonella typhimurium*. Am. Soc. Microbiol.: Washington, DC, 1996. 1553–1569.

Chalup, M. S., and Laws, E. A. (1990). A test of the assumptions and predictions of recent microalgal growth models with the marine phytoplankter *Pavlova lutheri*. *Limnol. Oceanogr.* 35, 583–596. doi:10.4319/lo.1990.35.3.0583.

Healey, F. P. (1985). Interacting effects of light and nutrient limitation on the growth rate of *Synechococcus linearis* (Cyanophyceae). *J. Phycol.* 21, 134–146.

Rittmann, B. E., and McCarty, P. L. (2001). Environmental Biotechnology: Principles and Applications. McGraw-Hill: New York, NY.

Sakshaug, E., and Andersen, K. (1989). A steady state description of growth and light absorption in the marine planktonic diatom *Skeletonema costatum*. *Limnol. Oceanogr.* 34, 198–205.
