# Supplementary material for: A Mechanistic Model of Macromolecular Allocation, Elemental Stoichiometry, and Growth Rate in Phytoplankton
Source: Front Microbiol. 2020 Feb 28;11:86. doi: 10.3389/fmicb.2020.00086 (PMC7093025; doi:10.3389/fmicb.2020.00086)
Supplement: Supplementary file 3 [file Data_Sheet_3.pdf]

Supplementary Text:

A mechanistic model of macromolecular allocation, elemental stoichiometry and growth rate in phytoplankton

Keisuke Inomura, Anne Willem Omta, David Talmy, Jason Bragg, Curtis Deutsch and Michael J. Follows

Contents:

A brief review of physiological models of phytoplankton

A brief review of physiological models of phytoplankton

Here we briefly review models of phytoplankton physiology to date. A more comprehensive review is beyond the scope of this article and a stand-alone article would be timely. The earliest (and still often employed) models of phytoplankton physiology relate growth rates of phytoplankton to the external or internal concentrations of a single limiting resource (Monod, 1949; Caperon, 1968; Droop, 1968). The internal-stores approach has been extended to represent multiple elemental pools (Ågren, 2004; Flynn, 2008) and include dynamic representations of photo acclimation, temperature dependence to predict both growth rate and elemental composition (Geider et al., 1998). The interdependence of the intracellular flow of N and P on the pools of the other element is represented in the "chain model" (Pahlow and Oschlies, 2009).

In a ground-breaking model (Shuter, 1979) explicitly resolved four functional compartments (photosynthetic, biosynthetic, structural and storage) and optimized allocation of carbon to maximize growth rate. The strategy of allocation to functional or macromolecular pools connects elemental composition and physiological function (Sterner and Elser, 2002). It constrains important trade-offs and has been employed in a number of models to represent the dependence of phytoplankton growth and elemental stoichiometry on light, temperature, nutrient availability and nutritional strategy (Flynn, 2001, 2005; Klausmeier et al., 2004; Clark et al., 2013; Toseland et al., 2013; Daines et al., 2014; Talmy et al., 2014; Ghyoot et al., 2017; Moreno et al., 2018; Nicholson et al., 2018).

Allocation within the protein and resource pool can also provide constraints, for example trade-offs between resource capture and intracellular assimilation (Smith et al., 2009; Bonachela et al., 2013) has been applied in ocean models (Smith et al., 2016; Chen and Smith, 2018a, 2018b; Chen et al., 2019). A minimal model of heterotrophic bacteria based on RNA/protein ratios and gene expressions (Scott et al., 2010) successfully predicted limits to growth under different resource conditions and

(Burnap, 2015), adapted a protein allocation model of heterotrophic bacteria (Molenaar et al., 2009) to include phototrophy and reproduced observed protein allocation in *Synechocystis* (Jahn et al., 2018). Similarly an optimum resource allocation model with coarse-grained allocation of protein (Faizi et al., 2018), showed good agreement with another recent proteomic study of *Synechocystis* (Zavřel et al., 2019). The model is further developed to simulate the protein allocation, cellular growth and biomass production in a light limited chemostat (Faizi and Steuer, 2019).

Over the past decades, models of phytoplankton physiology have gradually resolved in greater detail and realism the internal allocation of resources between and within the macromolecular pools of the cell. In the past this has typically been achieved in a rather abstract manner, though recent models connect more closely to measurable pools, including modern proteomics. This is the foundation of the approach which we adopt here. We note that genome scale modeling approaches (e.g. Flux Balance Analysis) represent microbial physiology as an outcome of thousands of cellular reactions (Orth et al., 2010), with the assumption that some aggregate outcome (often growth) is optimized. These can also be constrained by the notion of allocation (Müller et al., 2015; Rugen et al., 2015; Reimers et al., 2017) and are complimentary to the still aggregated allocation models of the type we present, which are more tractable for many practical applications.

References

Ågren, G. I. (2004). The C:N:P stoichiometry of autotrophs - Theory and observations. *Ecol. Lett.* 7, 185–191. doi:10.1111/j.1461-0248.2004.00567.x.

Bonachela, J. A., Allison, S. D., Martiny, A. C., and Levin, S. A. (2013). A model for variable phytoplankton stoichiometry based on cell protein regulation. *Biogeosciences* 10, 4341–4356. doi:10.5194/bg-10-4341-2013.

Burnap, R. L. (2015). Systems and photosystems: cellular limits of autotrophic productivity in cyanobacteria. *Front. Bioeng. Biotechnol.* 3, 1. doi:10.3389/fbioe.2015.00001.

Caperon, J. (1968). Population growth response of *Isochrysis galbana* to nitrate variation at limiting concentrations. *Ecology* 49, 866–872.

Chen, B., and Smith, S. L. (2018a). CITRATE 1.0: Phytoplankton continuous trait-distribution model with one-dimensional physical transport applied to the North Pacific. *Geosci. Model Dev.* 11, 467–495. doi:10.5194/gmd-11-467-2018.

Chen, B., and Smith, S. L. (2018b). Optimality-based approach for computationally efficient modeling of phytoplankton growth, chlorophyll-to-carbon, and nitrogen-to-carbon ratios. *Ecol. Modell.* 385, 197–212. doi:10.1016/j.ecolmodel.2018.08.001.

Chen, B., Smith, S. L., and Wirtz, K. W. (2019). Effect of phytoplankton size diversity on primary productivity in the North Pacific: trait distributions under environmental variability. *Ecol. Lett.* 22, 56–66. doi:10.1111/ele.13167.

Clark, J. R., Lenton, T. M., Williams, H. T. P., and Daines, S. J. (2013). Environmental selection and resource allocation determine spatial patterns in picophytoplankton cell size. *Limnol. Oceanogr.* 58, 1008–1022. doi:10.4319/lo.2013.58.3.1008.

Daines, S. J., Clark, J. R., and Lenton, T. M. (2014). Multiple environmental controls on phytoplankton growth strategies determine adaptive responses of the N:P ratio. *Ecol. Lett.* 17, 414–425. doi:10.1111/ele.12239.

Droop, M. R. (1968). Vitamn B_{12} and marine ecology. IV. The kinetics of uptake, growth and inhibition in *Monochrysis Lutheri*. *J. Mar. Biol. Assoc. United Kingdom* 48, 689–733.

Faizi, M., and Steuer, R. (2019). Optimal proteome allocation strategies for phototrophic growth in a light-limited chemostat. *Microb. Cell Fact.* 18, 165. doi:10.1186/s12934-019-1209-7.

Faizi, M., Zavřel, T., Loureiro, C., Červený, J., and Steuer, R. (2018). A model of optimal protein allocation during phototrophic growth. *BioSystems* 166, 26–36. doi:10.1016/j.biosystems.2018.02.004.

Flynn, K. J. (2001). A mechanistic model for describing dynamic multi-nutrient, light, temperature interactions in phytoplankton. *J. Plankton Res.* 23, 977–997.

Flynn, K. J. (2005). Modelling marine phytoplankton growth under eutrophic conditions. *J. Sea Res.* 54, 92–103. doi:10.1016/j.seares.2005.02.005.

Flynn, K. J. (2008). The importance of the form of the quota curve and control of non-limiting nutrient transport in phytoplankton models. *J. Plankton Res.* 30, 423–438. doi:10.1093/plankt/fbn007.

Geider, R. J., Macintyre, H. L., and Kana, T. M. (1998). A dynamic regulatory model of phytoplanktonic acclimation to light, nutrients, and temperature. *Limnol. Oceanogr.* 43, 679–694.

Ghyoot, C., Flynn, K. J., Mitra, A., Lancelot, C., and Gypens, N. (2017). Modeling plankton mixotrophy: A mechanistic model consistent with the Shuter-type biochemical approach. *Front. Ecol. Evol.* 5, doi: 10.3389/fevo.2017.00078 Modeling. doi:10.3389/fevo.2017.00078.

Jahn, M., Vialas, V., Karlsen, J., Maddalo, G., Edfors, F., Forsström, B., et al. (2018). Growth of cyanobacteria is constrained by the abundance of light and carbon assimilation proteins. *Cell Rep.* 25, 478–486. doi:10.1016/j.celrep.2018.09.040.

Klausmeier, C. A., Litchman, E., Daufresne, T., and Levin, S. A. (2004). Optimal nitrogen-to-phosphorus stoichiometry of phytoplankton. *Nature* 429, 171–174. doi:1.1029/2001GL014649.

Molenaar, D., Berlo, R. Van, and Ridder, D. De (2009). Shifts in growth strategies reflect tradeoffs in cellular economics. *Mol. Syst. Biol.* 5. doi:10.1038/msb.2009.82.

Monod, J. (1949). The growth of bacterial cultures. *Ann. Rev. Mar. Sci.* 3, 371–394.

Moreno, A. R., Hagstrom, G. I., Primeau, F. W., Levin, S. A., and Martiny, A. C. (2018). Marine

phytoplankton stoichiometry mediates nonlinear interactions between nutrient supply, temperature, and atmospheric CO_2. *Biogeosciences* 15, 2761–2779.

Müller, S., Regensburger, G., and Steuer, R. (2015). Resource allocation in metabolic networks: Kinetic optimization and approximations by FBA. *Biochem. Soc. Trans.* 43, 1195–1200. doi:10.1042/BST20150156.

Nicholson, D. P., Stanley, R. H. R., and Doney, S. C. (2018). A phytoplankton model for the allocation of gross photosynthetic energy including the trade-offs of diazotroophy. *J. Geophys. Res. Biogeosciences* 123, 1796–1816. doi:10.1029/2017JG004263.

Orth, J. D., Thiele, I., and Palsson, B. Ø. (2010). What is flux balance analysis? *Nat. Biotechnol.* 28, 245–248. doi:10.1038/nbt.1614.

Pahlow, M., and Oschlies, A. (2009). Chain model of phytoplankton P, N and light colimitation. *Mar. Ecol. Prog. Ser.* 376, 69–83. doi:10.3354/meps07748.

Reimers, A.-M., Knoop, H., Bockmayr, A., and Steuer, R. (2017). Cellular trade-offs and optimal resource allocation during cyanobacterial diurnal growth. *Proc. Natl. Acad. Sci.* 114, E6457–E6465. doi:10.1073/pnas.1617508114.

Rugen, M., Bockmayr, A., and Steuer, R. (2015). Elucidating temporal resource allocation and diurnal dynamics in phototrophic metabolism using conditional FBA. *Sci. Rep.* 5, 1–16. doi:10.1038/srep15247.

Scott, M., Gunderson, C.W., Mateescu, E. M., Zhang, Z., and Hwa, T. (2010). Interdependence of cell growth and gene expression: Origins and consequences. *Science* 330, 1099–1103.

Shuter, B. (1979). A model of physiological adaptation in unicellular algae. *J. Theor. Biol.* 78, 519–552. doi:10.1016/0022-5193(79)90189-9.

Smith, S. L., Pahlow, M., Merico, A., Acevedo-Trejos, E., Sasai, Y., Yoshikawa, C., et al. (2016).

Flexible phytoplankton functional type (FlexPFT) model: Size-scaling of traits and optimal growth. *J. Plankton Res.* 38, 977–992. doi:10.1093/plankt/fbv038.

Smith, S. L., Yamanaka, Y., Pahlow, M., and Oschlies, A. (2009). Optimal uptake kinetics: Physiological acclimation explains the pattern of nitrate uptake by phytoplankton in the ocean. *Mar. Ecol. Prog. Ser.* 384, 1–12. doi:10.3354/meps08022.

Sterner, R. W., and Elser, J. J. (2002). Ecological Stoichiometry: the Biology of Elements from Molecules to the Biosphere. Princeton University Press: Princeton, NJ.

Talmy, D., Blackford, J., Hardman-Mountford, N. J., Polimene, L., Follows, M. J., and Geider, R. J. (2014). Flexible C : N ratio enhances metabolism of large phytoplankton when resource supply is intermittent. *Biogeosciences* 11, 4881–4895. doi:10.5194/bg-11-4881-2014.

Toseland, A., Daines, S. J., Clark, J. R., Kirkham, A., Strauss, J., Uhlig, C., et al. (2013). The impact of temperature on marine phytoplankton resource allocation and metabolism. *Nat. Clim. Chang.* 3, 979–984. doi:10.1038/nclimate1989.

Zavřel, T., Faizi, M., Loureiro, C., Poschmann, G., Stühler, K., Sinetova, M., et al. (2019). Quantitative insights into the cyanobacterial cell economy. *Elife* 8. doi:10.7554/elife.42508.
